# Advanced Materials for Biologics Delivery to Brain Tumors

**DOI:** 10.1002/adma.74760

**Published:** 2026-08-29

**Authors:** Yuran Feng, Yuxue Cao, Maria Kavallaris, Taskeen Janjua, Amirali Popat

**Affiliations:** ^1^ School of Pharmacy and Pharmaceutical Science The University of Queensland Brisbane Queensland Australia; ^2^ Drug Delivery, Disposition and Dynamics Monash Institute of Pharmaceutical Sciences Monash University Melbourne Victoria Australia; ^3^ Children's Cancer Institute at Minderoo Children’s Comprehensive Cancer Centre Sydney New South Wales Australia; ^4^ Australian Centre for NanoMedicine UNSW Sydney Sydney New South Wales Australia; ^5^ School of Clinical Medicine UNSW Medicine & Health UNSW Sydney Sydney New South Wales Australia

**Keywords:** biologics, brain drug delivery, brain tumor, invasive drug delivery, noninvasive drug delivery

## Abstract

Brain cancer remains a major global health challenge due to its high morbidity and mortality. Current standard therapies are often limited by incomplete tumor resection, systemic toxicity, and the inability to effectively penetrate the blood‐brain barrier (BBB), which restricts drug delivery to the tumor site. These challenges underscore the urgent need for innovative therapeutic strategies that can overcome these barriers and improve treatment outcomes. Biological materials encompass a diverse range of molecules, from small peptides to monoclonal antibodies, either derived from living organisms or synthetically synthesized. Unlike traditional therapies, biologics provide a more targeted approach, including specific tumor targeting, immune system modulation, and regenerating damaged cells. Although biologics offer significant promise for brain cancer treatment, major challenges associated with blood–brain and tumour barriers continue to limit their translation, and comprehensive reviews bridging therapeutic advances with delivery technologies remain scarce. While individual biologic classes have been extensively studied, a unified assessment of these modalities and their delivery optimization is still needed. This review addresses the current gap by critically examining recent advances in biologic therapies and evaluating emerging invasive and non‐invasive delivery strategies to enhance their therapeutic efficacy in brain cancer.

## Graphical abstract

Material innovation is central to unlocking the therapeutic potential of biologics against many central nervous system diseases, including brain cancer. By engineering carriers with controlled transport, targeting, and release properties, advanced materials can overcome the blood–brain barrier and tumor microenvironment, improving the delivery of antibodies, nucleic acids, peptides, and cell‐based therapies for precision neuro‐oncology.

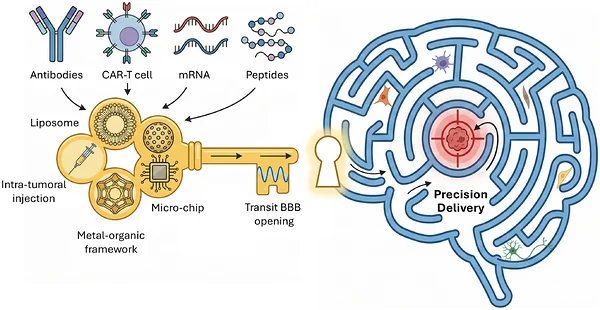

## 1 Introduction

Biological therapeutics, also referred to as biologics, represent a promising new frontier in materials. They are generally high molecular weight and structurally complex materials that act at the genetic or molecular level. This diverse class includes antibodies, proteins, vaccines, blood and blood components, recombinant therapeutic proteins, nucleic acids, and cell‐based therapies. According to the U.S. Food and Drug Administration (FDA), biologicals are defined as products that are isolated from a variety of natural sources, such as bacterial, yeast, plant, or animal cells, or produced using biotechnology and other cutting‐edge technologies [1]. Unlike conventional small molecule drugs (< 500 Da) which are usually chemically defined molecules with simple structures, biologics consist of intricate, heterogeneous mixtures that are challenging to fully characterize [1]. The rapid growth in the development of biological therapeutics over the last two decades has irrevocably changed the landscape of drug discovery and development. Biologics now account for approximately 40% of global pharmaceutical sales, with a market value of around USD 350 billion in 2023. This figure is projected to double to approximately USD 700 billion by 2032, driven by a compound annual growth rate of 7.8% [2, 3]. Additionally, nearly half of the annually approved drugs by regulatory agencies, such as the FDA and European Medicines Agency, have been biologics (Figure 1). They have emerged as a new generation of therapeutics due to their unique advantages over conventional treatments, including higher specificity for tumor‐associated antigens, reduced off‐target toxicity, and the ability to harness the immune system to selectively target and eliminate tumor cells. Moreover, biologics can bypass chemoresistance and reshape the tumor microenvironment, extending their therapeutic potential to multiple disease types, including brain cancer.

**FIGURE 1:**
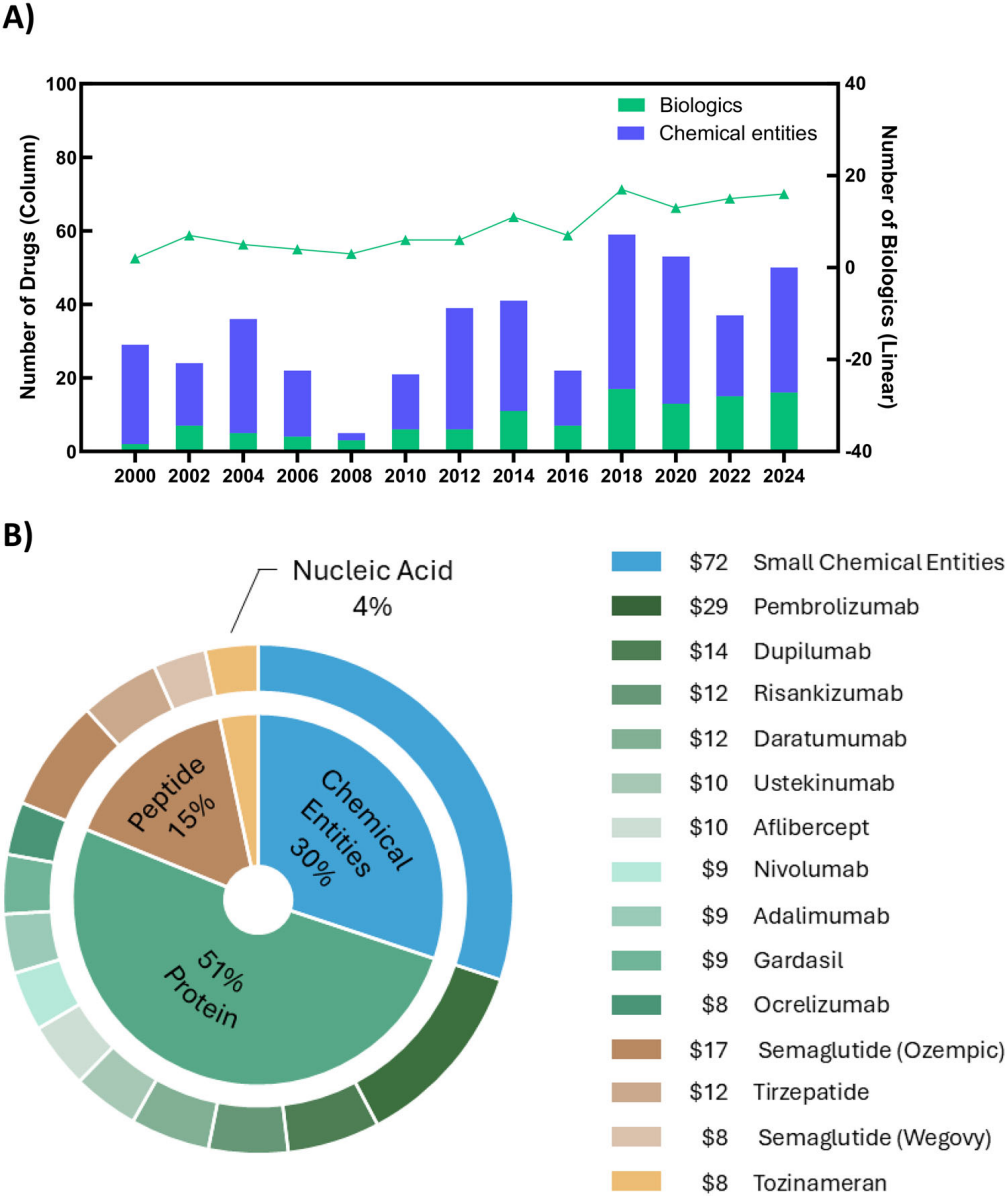
(A) New chemical entities and biologics approved by the FDA over the past 20 years. Reproduced from [23]. The number of biologics approved by the FDA each year shows an upward trend, indicating biologics are receiving increasing attention in research and development. (B) Total 2024 sales of the top 20 pharmaceuticals by drug class (USD, millions). Despite fewer approvals, biologics represent a disproportionately large share of the pharmaceutical market, with higher sales revenue and average daily cost (≈2× more expensive than small molecules [24]).

Brain cancer in both children and adults remains one of the most challenging malignancies with high morbidity and mortality rates worldwide. According to the Global Burden of Disease study, brain and central nervous system cancer accounted for more than 263,800 deaths globally in 2023 [4, 5]. Primary malignant brain tumors encompass a diverse group of diseases, including glioblastoma (GBM), diffuse midline glioma (DMG), medulloblastoma, anaplastic astrocytoma, and aggressive meningiomas [6]. Collectively, these tumors are characterized by their infiltrative growth, therapeutic resistance, and poor long‐term survival rates. Among adults, glioblastoma (IDH‐wildtype) is the most common primary brain tumor, accounting for over 50% of cases, with an annual incidence of approximately 3–5 cases per 100,000 individuals [7, 8]. The highly infiltrative and aggressive nature leads to limited treatment options and dismal prognosis, with a median survival of 12–18 months despite receiving therapy [7, 8, 9]. Anaplastic astrocytoma and oligodendrogliomas represent lower‐grade gliomas that can progress to high‐grade disease, often with more indolent clinical courses but still associated with high recurrence rates [9, 10, 11]. Aggressive meningiomas, although locate extracerebral, can exhibit malignant behavior with local invasion and recurrence [12, 13]. In pediatric populations, DMG is uniformly fatal, with a median survival of under one year, whereas medulloblastoma, the most common malignant pediatric cerebellar tumor, exhibits molecular heterogeneity across subgroups that influences prognosis and treatment response [14, 15]. The aggressive nature and dismal survival outcomes underscore the critical need for more effective treatment strategies.

The current standard of care for GBM and other aggressive brain tumors typically involves maximal safe surgical resection, followed by radiotherapy and concurrent chemotherapy [16, 17]. These treatments can improve patient outcomes to some extent, but they fail to stop tumor progression. This limitation arises from pronounced tumor heterogeneity, the immunosuppressive tumor microenvironment, surgically inaccessible tumor locations, and the protective role of the BBB [17, 18]. While the BBB safeguards neuronal function by tightly regulating the exchange of nutrients and shielding the brain from toxins, it also limits the penetration of most therapeutic agents. Conventional chemotherapies face additional obstacles, including poor aqueous solubility, insufficient targeting ability, and limited infiltration into tumor cells [17, 18]. As a result, the prognosis for patients with aggressive malignant brain tumors, particularly GBM, remains poor and is treatment‐dependent. The reported median overall survival for GBM is 11 to 15 months, with a progression‐free survival of approximately 9.5 months [7, 19, 20, 21]. These limitations highlight the urgent need for novel therapeutic strategies that can overcome both biological and pharmacological barriers to treatment.

Biologics have emerged at the forefront of next‐generation brain cancer therapies, yet challenges in their development and application remain. These include the instability of proteins and peptides, inefficient intracellular delivery of nucleic acids, and limited viability and expansion of live cells [22]. To address these barriers, three core strategies are typically employed: modification of the biologic itself to improve stability and functionality, alteration of the tumor or delivery environment, and the development of advanced drug delivery platforms to enhance transport by controlling interactions between biologics and their microenvironment. Effective drug delivery is pivotal for maximizing the therapeutic impact of biologics, particularly in brain cancer. An ideal delivery route should be designed based on the specific properties of each biologic to achieve precise and efficient transport across the challenges to target tumor sites while preserving therapeutic efficacy.

In this review article, we evaluate the application of four main classes of biologics— peptides, nucleic acids, proteins, and cells in the treatment of various brain cancers. Preclinical research, clinical trials, and commercial products are discussed to provide an overview of the current therapeutic landscape. Additionally, we discuss the biological barriers limiting biologics delivery to brain tumors and explore advanced nanomaterial‐enabled strategies to enhance transport, targeting, and therapeutic efficacy. For each delivery route, we highlight how rational material design can overcome the intrinsic limitations of biologics and redefine the therapeutic landscape of brain cancer.

## 2 Biologics for Brain Cancer

Biologics have become a transformative approach in the treatment of brain cancer, offering clear advantages over traditional small‐molecule therapies [25]. Their key strength lies in the ability to selectively target molecules and pathways that drive tumor growth. This feature is particularly crucial in brain cancers, where conventional therapies often show limited efficacy and poor outcomes [26, 27, 28]. Chemotherapy and radiation therapies, while partially effective, often damage healthy brain tissue and cause debilitating side effects. In contrast, biologics offer high specificity, harnessing the immune system to recognize and eliminate cancer cells while also inducing immunological memory within the adaptive immune response [25, 29]. Biologics can be applied in immunotherapy, gene therapy, and personalized medicine, enabling therapies tailored to each patient's unique tumor profile while overcoming chemotherapy resistance developed by tumor cells [30, 31, 32]. As a result, biologics represent a rapidly growing area in brain cancer therapy, offering new hope for patients where traditional treatments have fallen short.

With this promising foundation, the next step is to explore the various types of biologics being used or investigated for brain cancer, ranging from monoclonal antibodies to chimeric antigen receptor T‐cell therapies, each offering unique mechanisms of action and specific advantages in tackling the complexities of brain tumors (Figure 2). Nucleic acid therapies enable precise modulation of gene expression but are limited by instability and rapid degradation. Peptides offer high specificity yet are often constrained by short half‐life. Protein‐based therapeutics provide potent, target‐specific effects, but their large size and susceptibility to enzymatic degradation or structural denaturation hinder effective delivery. Cell therapies can exert sustained therapeutic activity, but challenges in manufacturing, safety, and central nervous system trafficking remain major barriers. The specific advantages and limitations of these biologics are evaluated in depth in the subsequent sections.

**FIGURE 2:**
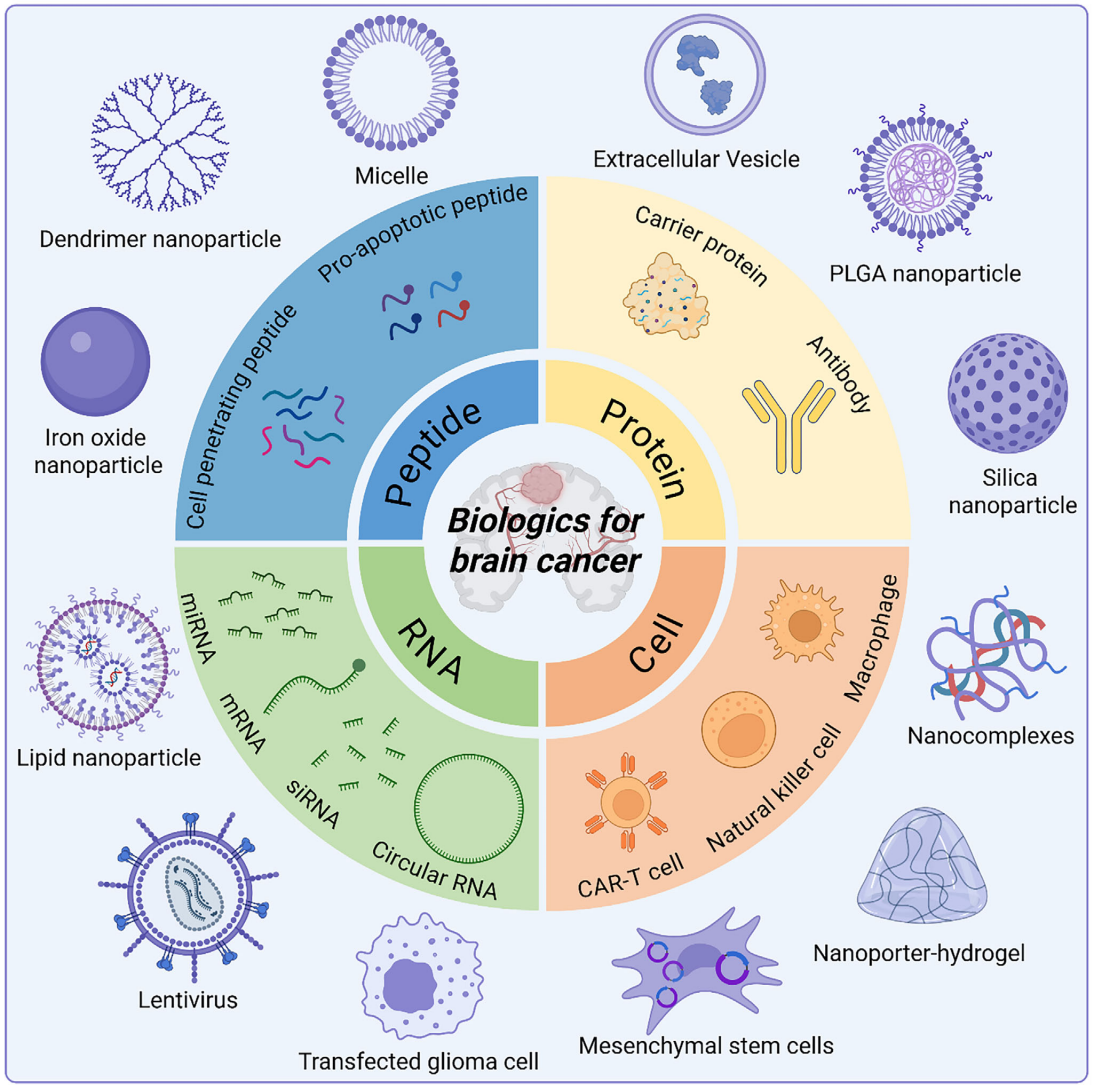
Biologics and advanced delivery platforms for brain cancer therapy. Major classes of biologics, including biosynthesized peptides, proteins, nucleic acids, and cell‐based therapies, are highlighted. These are presented alongside emerging delivery strategies, including lipid nanoparticles [33, 51, 53, 65], transfected cells [79, 82, 83, 84, 85], nanocomplexes [45, 121, 122], extracellular vesicle [38, 39, 40, 123, 124], dendrimers [41], mesenchymal stem cells [125], and PLGA nanoparticles [126, 127], designed to enhance stability, enable targeted delivery, and improve cellular internalization.

### 2.1 Nucleic Acid

Nucleic acid‐based therapies hold significant promise for the treatment of brain cancer, though several challenges must be addressed to fully realize their therapeutic potential. One primary obstacle is the inherent instability of nucleic acids. Naked nucleic acids, such as ribonucleic acid (RNA) and deoxyribonucleic acid (DNA), are highly susceptible to nuclease degradation in biological environments. This issue is further compounded by the human immune system, which efficiently identifies and eliminates foreign nucleic acids, reducing their therapeutic efficacy. Another critical challenge is the difficulty of traversing the BBB, a highly selective biological structure that restricts the entry of therapeutic agents into the brain. Even after crossing the BBB, nucleic acids need to be delivered to specific intracellular compartments to achieve their intended effects—to the cytoplasm of the cell for small interfering, messenger ribonucleic acid, or to the nucleus for antisense oligonucleotide, DNA, or CRISPR. This requires precise targeting and efficient cellular internalization while minimizing off‐target effects.

Progress in this field remained slow until the advent of nanotechnology since 2000, which has since revolutionized the landscape of therapeutic nucleic acid‐based therapy [28]. Since then, researchers have increasingly focused on employing non‐viral vectors, such as lipid nanoparticles [33], gold nanoparticles [34], polymeric nanoparticles [35, 36, 37], exosomes [38, 39, 40], and dendrimers [41], to enhance the stability of nucleic acids, prolong circulation time, and improve their delivery to neuronal and brain tumors like GBM. These nanocarriers protect nucleic acids from degradation, enable efficient BBB penetration, and facilitate targeted delivery to tumor cells, as summarized in Table 1. Additionally, the combination of advanced nanotechnologies with various administration strategies, such as convection‐enhanced delivery or intranasal routes, has further amplified the therapeutic potential. Encouraging outcomes from a phase 0 clinical trial (NCT03020017) underscore the promise of these advanced delivery approaches, paving the way for the transformation of nucleic acid‐based therapies in brain cancer treatment [34].

**TABLE 1:** Summary of therapeutic nucleic acid for treatment of brain cancer (organized by therapeutic category and publication year).

Therapeutic agent	Carrier	Model/tumor type	Therapeutic target	Administration	Key results	Year and Refs.
mRNA vaccine, PD‐1 antibody	Lipid nanoparticle	In vitro in HEK293T, DC2.4, and GL261 cell lines In vivo in BALB/c mice	Dendritic cells	IV	Completely eradicated glioma and reduced hepatotoxicity when combined with Anti‐PD‐1 therapy	2025 [51]
P53 mRNA, BRD4 siRNA	Fluorinated polyethyleneimine Nanocapsule + ApoE peptide	In vitro in GL261 glioma cell line In vivo in C57BL/6 mice	p53‐p21‐RB1 pathway, and PD‐L1	IV	Triggered the secretion of cytokines (IL‐10, TNF‐α, IFN‐γ) and improved mice survival	2025 [46]
Interleukin‐12 mRNA	Lipid nanoparticle + Tat peptide	In vitro in N2a, bEnd.3, and CT‐2A‐Luc glioma cell lines In vivo in C57BL/6J, C3H/HeJ, and Ai14 mice	Immune cells	IV	Achieved over a 50‐fold increase in mRNA translation within brain tissues compared to FDA‐approved Onpattro LNPS	2025 [53]
PTEN mRNA	Nanocomplex + ApoE peptide	In vitro in U87MG and patient‐derived CSC2 GSCs cell line In vivo in BALB/c mice	NA	IV	Exhibited a remarkable extension of the median survival time of mice with negligible side effects	2023 [45]
Interleukin‐12 mRNA	GL261 cell membrane coated CaCO_3_ nanoparticle + cRGD	In vitro in HepG2 cell, 4T1 cell, CT26 cell, and GL261 cell lines In vivo in C57BL/6 mice	Immune cells	IV	Tumor‐homing nanoparticles achieved necroptosis‐induced immune responses under ultrasound‐mediated release	2022 [48]
PTEN‐mRNA, TRAIL‐mRNA	NA	In vitro in DBTRG‐Luc and patient‐derived glioma cell line In vivo in DBTRG‐Luc xenograft nude mice	JNK pathway	IC	Synergistic mRNA‐based therapy significantly inhibited tumor growth in nude mice	2022 [43]
IFNα2, IL12 mRNA	NKG2D CAR T cells	In vitro in GL‐261, SMA‐560, and CT‐2A glioma cell lines In vivo in C57BL/6 mice	Glioma cells, lymphoid and myeloid cells	IT	Modified CAR T cells coexpressing IL12 and IFNα2 increased anti‐glioma activity by promoting a proinflammatory TME and mitigating T‐cell exhaustion	2022 [47]
TRAIL‐mRNA	NA	In vitro in 293T cell line In vivo in C57BL/6J mouse model	Death receptor 4, death receptor 5	IC	TransIT‐mRNA has a better effect than jetPEI in mRNA intracranial transfection	2022 [74]
PTEN mRNA	Exosome + CDX/CREKA targeting peptide	In vitro U87‐MG and HEK293T cell lines In vivo in BALB/C‐nu and C57BL/6 mice	NA	IV	Achieved up to 50‐fold more exosomes and a more than 10^3^‐fold increase in exosomal mRNA transcripts	2020 [38]
Cas9 mRNA, sgRNA	Lipid nanoparticle	In vitro in 0005 MG, HCT116, and A549 cells In vivo in 57BL/6JOlaHsd mice	CRISPR‐Cas9 gene	IC	Achieved therapeutic genome editing in both ovarian and orthotopic glioblastoma models	2020 [33]
siRNA	Bispecific antibody + PEGylated lipid nanoparticles	In vitro in H460, and CHP‐134 cell line In vivo in SCID‐beige mice	PLK1	IV	EGFR‐targeted siRNA nanoparticles enhanced PKL1 silencing in vitro and reduced neuroblastoma growth in vivo	2024 [65]
siRNA	Cholesterol‐modified T7 nanomicelle	In vitro in GL261, BV2, 293T cell lines In vivo in the GL261 xenograft mouse model	slit2 gene	IN	Promoting polarization of M2 subtype macrophages to M1 subtype macrophages Increased the proportion of effector T cells	2023 [75]
siRNA	Hyaluronic acid + DP7‐C peptide	In vitro in GL261, U87MG, U251, A172 and T98G glioma cell lines In vivo in C57BL/6 J mice	CD44	IN	Decrease tumor volume and extend the mice survival	2022 [62]
siRNA	Lipid‑calcium‐phosphate nanoparticles	In vitro in GBM ALTS1C1 cell line In vivo in C57BL/6JNarl mice	Immune checkpoint ligand PD‐L1	IV	Suppress GBM progression by silencing PD‐L1 expression and activating intratumoral cytotoxic T cells	2022 [64]
siRNA	Exosome + Angiopep‐2	In vitro in U87MG cell line In vivo in BALB/c nude mice	STAT3	IV	Exhibited efficient BBB penetration, GBM targeting, and inhibition of GBM growth	2022 [39]
siRNA + TMZ	Peptide‐modified exosomes	In vitro in L02, U87, bEnd.3, U251cell lines In vivo in the BALB/c athymic mouse model	STAT3	IV	Restored TMZ sensitivity and achieved synergistic anti‐tumor effect	2022 [40]
siRNA LSINCT5	Dendrimer nanoparticle + peptides tLyp‐1 + anti‐NKG2A monoclonal antibody	In vitro in U87 cell line In vivo in xenograft nude mice	miR‐451/PI3K pathway	IV	Inhibited glioma cell growth by upregulating T and NK cells activity, further extending mice medium survival	2021 [41]
siRNA	Lipid‐polymer hybrid nanoparticle	In vitro in GL291 MG cell line; in vivo in C57BL/6J mice	Smoothened‐activated sonic hedgehog subgroup	FUS + microbubbles	Achieved over 10‐fold increase in siRNA delivery; reduced SMO protein level, resulting in significantly increased tumor cell apoptosis in vivo	2021 [76]
siRNA + TMZ	Iron oxide nanoparticle + targeting chlorotoxin	In vitro in A1235, U87, U118 glioma cell lines; in vivo in C57BL/6J WT and GBM6 mouse model	O6‐methylguanine‐DNA methyltransferase	IV	Increased apoptosis of GBM stem‐like cells and significantly prolonged lifespan	2021 [73]
siRNA	Cationic nanoemulsion	In vitro in C6 glioma cell line; in vivo in wistar rats	CD73	IN	Delivered siRNA to the brain by IN administration, leading to decreased tumor CD73 expression	2020 [72]
siRNA	Nanocapsules + Angiopep‐2	In vitro in the U87 MG cell line; in vivo in Xenografts U87MG mice	PLK1 mRNA	IV	Improved BBB penetration, GBM accumulation and retention remarkably improved survival in mice	2020 [63]
siRNA + Cisplatin	Iron oxide nanoparticles + folate	In vitro in U87 MG and P3#GBM cell lines; in vivo in the U87 MG xenograft nude mice	Glutathione peroxidase 4	IP	Induced ferroptosis and apoptosis in glioma cells to achieve antitumor effects	2020 [68]
siRNA	Bioreducible polymeric nanoparticles	In vitro in GBM patient‐derived cell line; in vivo in athymic nu/nu mice	Robo1, YAP1, NKCC1, EGFR, and survivin	IT	Verified the role of different genes in modulating tumor behavior	2019 [36]
Long Non‐coding RNA	NA	In vitro in H3.3 K27M DIPG cell line	H19	NA	H19 expression was elevated in DIPG compared with normal brain tissue, and its knockdown reduced cell proliferation and survival in DIPG cell	2021 [77]
hsa_circ_0004872	NA	In vitro in IOMM‐Lee and CH157‐MN meningioma cell lines; in vivo in BALB/c nude mice	miR‐190a‐3p	IV	Upregulation of Hsa_circ_0004872 inhibits meningioma cell proliferation and promotes apoptosis	2024 [78]
Circ_0001162	Transfected LN18, A172 cells	In vitro in LN18 and A172 glioma cell lines; in vivo in BALB/c nude mice	miR‐936/ERBB4 axis	NA	Improved glioma cell proliferation, migration and invasion	2021 [79]
Circ‐CDC45	Plasmid	In vitro in U87MG, U118, U251, and LN229 glioma cell lines	miR‐516b and miR‐527	NA	Facilitated proliferation and invasion of glioma cells	2020 [80]
circRNA *CDR1as* + Doxorubicin or VP16	Transfected cells	In vitro in HCT116, U87 MG, LN229 cell lines; in vivo in BALB/c nude mice	p53	NA	*CDR1as* directly binds to p53, preventing its interaction with MDM2 and inhibiting tumorigenesis	2020 [81]
CircPOSTN	Transfected LN229 cells	In vitro in NHA and U251, LN229 glioma cell lines; in vivo in BALB/c nude mice	miR‐361‐5p/TPX2 axis	NA	CircPOSTN promoted glioma progression by sponging miR‐361‐5p, which stimulates proliferation and glycolysis while inhibiting apoptosis	2020 [82]
circ‐MAPK4	Transfected U138 cells	In vitro in U138, U373, and U87 glioma cell lines; in vivo in BALB/c nude mice	p38/MAPK pathway	IC	Circ‐MAPK4 can promote tumorigenesis by inhibiting cell apoptosis	2020 [83]
Circ‐TTBK2	Transfected cells	In vitro in LN229 and U251 glioma cell lines; in vivo in Balb/c nude mice	miR‐761/ITGB8 axis	NA	Promoted glioma cell proliferation, invasion, and inhibited ferroptosis by sponging miR‐761 and targeting ITBG8	2020 [84]
Circ‐FBXW7	Transfected U251, U373 cells	In vitro in U251 and U373 cell lines; in vivo in the BALB/c‐nu mouse model	c‐Myc	IC	Inhibited cancer cell proliferation and cell cycle progression	2018 [85]
Hsa‐miR‐483‐5p mimic	NA	In vitro in meningioma‐derived C157‐MN and F5 cell lines	miR‐483‐5p/IGF‐2	NA	Meningiomas exhibit grade‐dependent overexpression of miR‐483‐5p; inhibition of either miR‐483‐5p or its target, IGF‐2, suppressed tumor growth	2023 [86]
Anti‐microRNA‐21 oligonucleotides	Co‐micelles + T7 peptide	In vitro in C6 glioma cell line; in vivo in Sprague Dawley rats	miRNA‐21	IN	Downregulation of miR‐21 triggered programmed cell death in tumor tissue	2023 [87]
miR‐153‐5p	NA	In vitro in IOMM‐Lee meningioma cell line	MEK/ERK	NA	Inhibited the proliferation, invasion, and migration of meningioma	2022 [88]
lentivirus‐hsa‐miR‐451	lentivirus	In vitro in U251, U87 cell lines; in vivo in BALB/c‐A nude mice	Targeting CAB39, inhibiting PI3K/Akt/Snail signaling pathway	IP	Targeting CAB39 suppresses glioma growth by inhibiting the PI3K/Akt/Snail signalling axis	2021 [89]
miR‐362 mimics	NA	In vitro in NHA and A172 GBM cell lines	MAPK1	NA	MiR‐362 inhibited GBM cell proliferation and metastasis by targeting MAPK1	2020 [90]
miR‐106b‐5p mimics	NA	In vitro in THP‐1, Raw264.7, HEK293T cell lines; in vivo in C57BL/6J mouse model	IRF1/IFN‐β pathway	IP	miR‐106b‐5p promoted M2 macrophage polarization to M1 by inhibiting IRF1/IFN‐β signaling	2020 [91]
miR‐128‐3p mimics	NA	In vitro in U87, T98, LN229, U251 and A172 cell lines; in vivo in SCID mouse model	c‐Met	NA	Sensitized glioma cells to TMZ by inhibiting c‐Met expression and suppressing the epithelial‐mesenchymal transition	2020 [92]
miR‐503 mimics	NA	In vitro in HCMEC/D3, A172‐MG, and U251‐MG cell lines	LRIG2	NA	LRIG2 inhibition downregulates VEGFA expression, thereby suppressing angiogenesis and glioma progression	2019 [93]
miR‐200a	Plasmid‐transfected glioma cells	In vitro in A172, U251MG, patient‐derived GBM cell lines; in vivo in athymic nude mouse model	Akt	IC	Downregulation of Gαi1 expression triggers Akt inactivation, thereby inhibiting glioma progression	2018 [94]
lentivirus‐hsa‐miR‐451	lentivirus	In vitro in LN229, U87 cell lines; in vivo in the U87 xenograft BALB/c‐A nude mice	Targeting CAB39, modulating mTOR/HIF‐1α/VEGF signaling pathway	NA	Upregulated miR‐451 inhibited glioma cell growth and invasion	2018 [95]
Antisense oligonucleotides	Lipid polymer micelle + Ang‐2	In vitro in the LN229 GBM cell line; in vivo in the BALB/c nude mice	miRNA‐21	Oral	Oral administration successfully activated the pro‐apoptotic protein p53 and Caspase‐3 to inhibit tumor progression	2024 [96]
Anatagomir‐21	RAGE‐antagonist peptide	In vitro in C6 GBM cell line; in vivo in Sprague Dawley mice	miRNA‐21	IN	Reducing intratumoral miR‐21 levels upregulates pro‐apoptotic gene expression, thereby inducing tumor cell apoptosis	2021 [97]
Plasmid DNA	Polymeric nanoparticle	In vitro in U87 glioma and B16F1ova melanoma cell lines; in vivo in the C57BL/6 mouse model	Tumors	MRI‐guided FUS + IV + microbubbles	Systemic delivery of Cy5‐labeled plasmid DNA achieved a 100% increase in intratumoral dispersion	2020 [98]

Abbreviations: CED, convection‐enhanced delivery; IV, intratumoral injection; IT, intratumoral injection; IP, intraperitoneal injection; IC, intracranial injection; IN, intranasal injection; LRIG2, immunoglobulin‐like domains protein 2; STAT3, signal transducer and activator of transcription 3; U251‐TR, TMZ resistant U251; RAGE, receptor for advanced glycation end‐products; TRAIL, TNF‐related apoptosis‐inducing ligand; DBTRG, Human glioblastoma cell line; ERBB4, ErbB2 receptor tyrosine kinase 4; TTBK2, Tau tubulin kinase 2; ITGB8, integrin subunit beta 8; NHA, normal human astrocytes; Robo1, Roundabout homolog 1; YAP1, Yes‐associated protein 1; NKCC1, sodium‐potassium‐chloride cotransporter; EGFR, endothelial growth factor receptor; PLK1, polo‐like kinase 1; MRI, magnetic resonance image; CAR, chimeric antigen receptors; NA, not applicable; PD‐L1, programmed death‐ligand 1; Cas9, CRISPR‐associated protein 9; HCMEC/D3, human cerebral microvascular endothelial cell line; MAPK1, mitogen‐activated protein kinase 1.

#### 2.1.1 Messenger RNA (mRNA)

Messenger RNA (mRNA) has been widely investigated as a promising tool for cancer therapy due to its critical role in protein expression and several inherent advantages. Tumors are characterized by genetic mutations and aberrant protein expression. Introducing synthetic mRNA into the cytoplasm enables the production of therapeutic proteins or peptides without requiring nuclear entry, thereby avoiding the risk of genomic integration associated with DNA‐based approaches. Compared with cell‐based therapies or monoclonal antibodies, mRNA therapeutics feature a simpler, highly flexible, and scalable manufacturing process, enabling rapid design and modification for personalized treatment. By harnessing the patient's own cellular machinery to produce therapeutic protein efficiently, mRNA therapeutics eliminate the need for complex protein manufacturing and the stringent handling conditions required for live‐cell‐based systems [42].

mRNA therapeutics are emerging as a versatile platform for brain cancer treatment, with potential applications across several complementary mechanisms. First, mRNA can restore the expression of tumor suppressor proteins that are frequently lost or mutated in malignant cells, such as p53 or PTEN, thereby restoring cell‐cycle control and promoting apoptosis [43, 44, 45, 46]. Second, mRNA can modulate the tumor microenvironment by encoding immunostimulatory cytokines, such as Interleukin‐12 and Interleukin‐21. These cytokines activate CD8+ cytotoxic T cells and natural killer cells against tumor tissues [47, 48, 49]. Beyond direct tumor modulation, mRNA vaccines have gained increasing attention in cancer immunotherapy, particularly as mRNA vaccines. In this approach, mRNA encoding tumor‐associated antigens or patient‐specific neoantigens is delivered to antigen‐presenting cells, especially dendritic cells. These cells then translate the encoded proteins and present the antigenic peptides to T lymphocytes, thereby priming cytotoxic CD8^+^ T cells to recognize and eliminate tumor cells [50, 51].

However, efficient intracellular delivery across brain remains a major challenge for mRNA therapeutics. To meet the therapeutic purpose, mRNA must reach the cytoplasm of target cells and express sufficient functional proteins. However, naked mRNA is highly susceptible to nuclease degradation and lacks tissue specificity. Protective delivery platforms are essential to ensure stable and efficient transport to the target site [33]. Lipid nanoparticles (LNPs) are FDA–approved nonviral vectors for mRNA delivery, enabling safe and efficient transport of mRNA across various biological barriers. Their ionizable lipid components electrostatically complex with negatively charged mRNA, protect it from degradation, and facilitate cellular uptake [52, 53]. To enhance brain delivery, BBB‐penetrating ligands are introduced to LNPs. For example, Tat‐modified LNPS increased mRNA translation in brain tissue by ∼50‐fold compared with FDA‐approved Onpattro lipid nanoparticles [53]. In another study, peptide‐functionalized LNPs targeting receptors enriched on brain endothelial cells and neurons (e.g., RVG29 and T7) significantly improved neuronal transfection [54]. After reaching target cells, the designed positively charged ionizable lipid nanoparticles promote cellular uptake through electrostatic interactions with the negatively charged cell membrane, facilitating endocytosis and subsequent release of the encapsulated mRNA [55].

Extracellular vesicles (EVs) have emerged as attractive nucleic acid carriers due to their inherent biocompatibility, low immunogenicity, favorable pharmacokinetics, and natural ability to traverse the BBB. However, loading exogenous nucleic acids, particularly large mRNAs with longer sequences, into cell‐secreted exosomes often resulted in low yields. Yang et al. addressed this challenge by developing a cellular nanoporation method that significantly enhanced the production of therapeutic mRNA‐loaded exosomes [38]. This strategy achieved a 50‐fold increase in exosome yield and more than a 10^3^‐fold enhancement in exosomal mRNA transcripts than bulk electroporation and other conventional strategies [38]. To further improve antitumor efficacy, microfluidic electroporation combining nano‐ and millisecond pulses was used to generate large quantities of IFN‐γ mRNA–loaded small EVs expressing CD64. Intravenous administration of these engineered EVs preferentially accumulated in GBM cells and elicited potent antitumor activity in vivo [56].

Other promising strategies have also been explored. GL261 cell membrane‐coated CaCO_3_ nanoparticles have been used to deliver interleukin‐12 mRNA to allow ultrasound‐triggered release and induce a necroptosis‐associated immune response [48]. Multifunctional CAR T cells co‐expressing Interleukin‐12 and Interferon‐alpha 2 demonstrated enhanced anti‐glioma activity in preclinical models [47]. Apolipoprotein E peptide‐grafted nanocomplexes delivering PTEN mRNA restored tumor‐suppressor function and prolonged survival in tumor‐bearing mice [45]. Similarly, ApoE peptide‐grafted nanocapsule co‐deliverying p53 mRNA and BRD4 siRNA to inhibit immune evasion and induce immunogenic cell death [46].

Recently, two new clinical trials have begun investigating mRNA‐based strategies for the treatment of brain tumors. In 2023, a first‐in‐human phase I trial (NCT05938387) was initiated to evaluate the CVGBM mRNA vaccine developed by CureVac in 37 patients with MGMT‐unmethylated glioblastoma. The vaccine is based on CureVac's RNActive platform and utilizes an mRNA construct with unmodified nucleotides, incorporating optimized noncoding elements shared with the company's second‐generation SARS‐CoV‐2 vaccine (CV2CoV). It is formulated in lipid nanoparticles derived from clinically validated systems previously used in clinical development of CVnCoV and related to those in approved mRNA vaccines [57]. Initial results indicate that the vaccine is generally well tolerated with an acceptable safety profile, while progression‐free and overall survival outcomes are under evaluation [58]. More recently, the PaedNEO‐VAX study has been initiated to investigate personalized mRNA vaccines for children and adolescents with relapsed or treatment‐resistant high‐grade brain tumors across multiple pediatric hospitals in Australia [59]. The four‐year, multi‐center trial will enroll approximately 70 patients and uses whole‐exome and tumor genome sequencing to identify patient‐specific tumor antigens for the generation of bespoke mRNA vaccines. The study targets aggressive pediatric brain cancers, including diffuse midline glioma, medulloblastoma, and ependymoma, all of which currently have limited effective treatment options. Vaccine production is undertaken through Australian manufacturing partnerships, enabling rapid translation of individualized formulations. Trial site activation commenced in 2026, marking the start of patient recruitment and clinical implementation. Early clinical evaluation focuses on safety, feasibility, and immune activation, with subsequent phases expected to assess effects on tumor control and survival outcomes.

Clinical translation of mRNA therapeutics for brain tumors is now rapidly advancing beyond preclinical exploration, supported by the emergence of multiple first‐in‐human and personalized vaccine trials (CVGBM mRNA vaccine and PaedNEO‐VAX). While challenges remain in delivery efficiency, molecular stability, and immune regulation, these barriers are being actively addressed through next‐generation nanocarrier engineering and improved delivery routes, including intranasal and targeted nanoparticle systems. The initiation of ongoing clinical programmes marks a pivotal shift toward real‐world application, indicating that mRNA‐based strategies are no longer theoretical but are entering early stages of clinical validation for aggressive brain tumors. Continued optimization of delivery platforms and immune control will be critical to fully realize their therapeutic potential in neuro‐oncology.

#### 2.1.2 Small Interfering RNA (siRNA)

Small interfering RNA (siRNA) is a type of double‐stranded RNA molecule that can silence gene expression through RNA interference. Once delivered into the cytoplasm, siRNA incorporates into the RNA‐induced silencing complex, which subsequently triggers mRNA degradation, thereby inhibiting protein translation and achieving efficient gene silencing [60]. With high specificity and low toxicity, siRNA holds considerable promise for treating central nervous system diseases, including GBM [61]. However, clinical translation remains challenging due to siRNA's rapid degradation in the serum environment, limited cellular uptake, and restricted ability to cross the BBB.

To overcome these limitations, innovative drug delivery strategies have been extensively investigated. Nanomicelles [37, 62], nanocapsules [63], lipid nanoparticles [64, 65], polymeric nanoparticles [36, 66, 67], iron oxide nanoparticles [68, 69], silicon nanoparticles [70, 71], and exosomes [39] have been demonstrated to enhance the poor stability and extend the blood circulation of siRNA. For instance, a multifunctional single siRNA nanocapsule was designed with disulfide‐crosslinked polymer shells to protect siRNA from degradation and enable controlled release in response to high glutathione levels in the cytoplasm [63]. To overcome the restrictive nature of the BBB, functionalization of nanocarriers with targeting ligands is the most used strategy [39, 63, 68]. These ligands promote receptor‐mediated transcellular transport by binding to receptors that are overexpressed on the BBB. Beyond receptor‐mediated approaches, nitric oxide donors were alternative methods to transiently disrupt the BBB, have also been explored. For example, Nitric oxide donors were encapsulated within nanoparticles to temporarily increase BBB permeability for intracellular delivery of programmed death‐ligand 1 siRNA specifically into GBM cells [64]. In addition, alternative administration routes such as the intranasal pathway have also been explored to bypass the BBB [72]. A multifunctional core‐shell nanomicelle comprising a DP7‐C cell‐penetrating peptide has achieved efficient intranasal delivery of siRNA directly into the central nervous system (CNS) [62].

Given the versatility of siRNA‐based therapies, combination strategies have further expanded their therapeutic potential. Co‐administration of siRNA with chemotherapy agents can overcome drug resistance and amplify anti‐tumor effects. For example, silencing the DNA repair enzyme O^6^‐methylguanine‐DNA methyltransferase sensitizes GBM cells to temozolomide treatment [73]. This combination therapy remarkably suppressed tumor growth, promoted apoptosis, and prolonged survival in orthotopic GBM mouse models [73]. Beyond chemosensitization, siRNA‐mediated ferroptosis induction has also shown promise. Folate‐modified iron oxide nanoparticles co‐loaded with cisplatin and glutathione peroxidase 4‐targeting siRNA effectively triggered ferroptosis and apoptosis in vivo. Their formulation successfully inhibited tumor proliferation and extended survival in GBM‐bearing mice [68].

Inspired by these successes, the clinical translation of mRNA‐based therapeutics has advanced and now enters early‐phase trials (Table 2). In a phase 0 first‐in‐human trial (NCT03020017), gold nanoparticles were selected as the delivery platform for precise quantification of gold distribution in cells and tumors. Intravenous administration of RNA‐conjugated gold nanoparticles successfully reached the tumor site and reduced tumor‐associated Bcl‐2‐like protein 12 expression. The safety and tolerability were well demonstrated, with no grade 4 or 5 treatment‐related toxicities reported [34].

**TABLE 2:** Summary of clinical trials conducted using nucleic acids for treatment of brain cancer (organized by completion year).

Therapeutic agent	Category	Delivery approach	Application/indication	Status	NCT number
CVGBM	mRNA vaccine	IM	MGMT‐unmethylated GBM	Completed 2026	NCT05938387 (phase I)
SiRNA	Nucleic acid	IV + Gold nanoparticle	Recurrent GBM	Completed 2020	NCT03020017 (phase I)

Abbreviations: IV, intravenous injection; IM, intramuscular injection; GBM, glioblastoma; MGMT, O‐6‐methylguanine‐DNA methyltransferase; NA, not applicable.

#### 2.1.3 Circular RNA (_Circ_RNA)

Circular RNAs (circRNAs) are a class of noncoding RNAs longer than 200 nucleotides, characterized by their covalently closed‐loop structures lacking 5′‐to‐3′ polarity. CircRNAs are highly stable, abundant, and widely distributed across various tissues, cell types, and biological fluids, making them easily detectable and ideal as biomarkers. Linear mRNAs are inherently unstable and prone to RNase degradation during storage, delivery, and transport [99]. Although nucleotide modifications to the mRNA backbone and untranslated regions can enhance stability, they also increase production costs and complexity in the manufacturing process [100]. In contrast, circRNAs exhibit superior intrinsic stability and can withstand room temperature storage and repeated freeze–thaw cycles [101, 102]. Unlike mRNA‐based therapies, which have high immunogenicity and the potential to trigger undesired cytotoxic responses [103, 104], circRNAs exhibit lower immunogenicity, offering a safer and more biocompatible alternative [105, 106]. By acting as miRNA sponges, interacting with proteins, or modulating parental gene expression, circRNAs play critical regulatory roles in cancer‐related processes, including cell proliferation, migration, invasion, and apoptosis. Emerging evidence indicates a strong correlation between circRNA and various cancers, with their potential in early screening and prognosis monitoring, particularly in astrocytoma, has been explored [82, 83, 107, 108, 109]. Depending on their specific functions within tumor biology, circRNAs may act as oncogenic drivers or tumor suppressors, positioning them as promising therapeutic targets to defeat brain cancers.

Oncogenic circRNAs, such as circPOSTN [82], circ‐MAPK4 [83], circTTBK2 [84], circ‐CDC45 [80], and circ_001162 [79], promote GBM progression through distinct mechanisms. For instance, circ‐MAPK4, as demonstrated by He et al., is upregulated in gliomas. This circRNA enhances cell survival and inhibits apoptosis by sponging miR‐125a‐3p, thereby modulating the p38/MAPK signaling pathway, as shown in BALD/c nude mice [83]. CircPOSTN was found to be overexpressed in glioma tissues and regulates TPX2 expression by sponging miR‐361‐5p. Knockdown of circPOSTN inhibited aerobic glycolysis and proliferation, while promoting apoptosis in glioma cells [82].

Conversely, tumor suppressor circRNAs, including circ‐FBXW7 [85] and CDR1as [81], hsa_circ_0004872 [78], inhibit glioma tumorigenesis through unique mechanisms. Hsa_circ_0004872 functions as a miRNA sponge to negatively regulate miR‐190a‐3p. Its overexpression suppresses meningioma cell proliferation while promoting apoptosis [78]. Circ‐FBXW7 and its protein product FBXW7‐185aa has been demonstrated to be abundant in normal brain tissues but downregulated in GBM. Upregulation of FBXW7‐185aa inhibits proliferation and cell cycle progression in cancer cells, suggesting circ‐FBXW7 as a potential therapeutic target and prognostic biomarker [85]. Similarly, expression of CDR1as decreases with increasing glioma grade and independently predicts overall survival, particularly in GBM patients. Rather than acting as a miRNA sponge, CDR1as binds to p53, preventing its ubiquitination and degradation by disrupting the p53/MDM2 interaction, consequently preserving p53 function and protecting cells from DNA damage in BALB/c nude mice in vivo [81].

CircRNAs exhibit remarkable stability and the capability to modulate key cancer‐related pathways, making them attractive candidates for glioma therapeutic development. Recent studies have demonstrated their potential as both diagnostic biomarkers and therapeutic targets. Continued investigation of their molecular mechanisms may reveal new avenues for the development of effective glioma treatments.

#### 2.1.4 MicroRNA (miRNA)

Advances in molecular biology have led to the discovery of novel therapeutic targets and diagnostic markers, offering a new avenue for improving the poor prognosis of brain cancers and enabling earlier diagnosis [110]. Among these, microRNA (miRNA)‐based therapy has emerged as a promising approach. The majority of the human genome is transcribed into non‐coding RNA, and miRNAs (18‐22 nucleotides in length) are the most extensively studied type [111]. MiRNA can regulate gene expression by binding to complementary sequences on target mRNAs, leading to mRNA degradation and translational repression. Beyond this, miRNAs also participate in more complex regulatory processes, including transcriptional activation, augmentation of protein translation, interactions with RNA‐binding proteins, and suppression of nuclear or mitochondrial transcripts [112]. Given their diverse regulatory functions, miRNAs can function as biomarkers for diagnosis, recurrence prediction, and assessment of tumor progression, particularly in meningioma and GBM [113, 114, 115]. Moreover, miRNAs can also act as either oncomiRs or tumor suppressors, playing critical roles in tumor progression. Consequently, therapeutic strategies may aim to restore the activity of downregulated tumor suppressor miRNAs or inhibit oncomiRs expression, providing a versatile approach to modulate tumorigenesis.

Restoration of tumor‐suppressive miRNA activity can be achieved using miRNA mimics, synthetic double‐stranded RNA molecules that replicate the sequence of endogenous miRNAs. These mimics are incorporated into the RNA‐induced silencing complex, effectively restoring the lost anti‐tumorigenic functions of the original miRNAs. Zhao et al. investigated the role of miR‐128‐3p mimics in enhancing temozolomide (TMZ) sensitivity. Typically downregulated in GBM, miR‐128‐3p was found to suppress GBM cell proliferation and invasion by downregulating epithelial–mesenchymal transition (EMT)‐related proteins. When co‐administered with TMZ in tumor‐bearing mice, miR‐128‐3p mimics were shown to remarkably reduce tumor volume and invasion profile [92]. miR‐200a is an anti‐Gαi1 microRNA that plays a regulatory role in glioma progression. Downregulation of miR‐200a in human gliomas leads to Gαi1 overexpression, triggering Akt pathway activation and promoting glioma cell proliferation [94]. For meningioma therapy, Chen et al. identified miR‐153‐5p and GAB1 as potential therapeutic and diagnostic targets. miR‐153‐5p is downregulated in meningioma tissues, and its restoration suppresses tumor cell growth by targeting GAB1 and inhibiting the MEK/ERK signaling pathway [88]. Interestingly, it has been reported that a single miRNA can target multiple genes, and multiple miRNAs can regulate a single gene [116, 117]. A study identified miR‐451 as a potential therapeutic target for glioma treatment. By targeting CAB39, miR‐451 inhibited the PI3K/Akt/Snail signaling pathway, thereby reducing epithelial‐mesenchymal transition and metastasis in glioma cells both in vitro and in vivo [89]. Working on the same miRNA, another study reported that miR‐451 inhibits glioma cell proliferation and invasion by suppressing the mTOR/HIF‐1α/VEGF signaling pathway. These findings underscore the dual role of miR‐451 in modulating critical pathways associated with tumor progression and metastasis, making it a promising target for glioma therapy [95].

In contrast, oncogenic miRNAs can be suppressed using targeted miRNA inhibitors, such as antisense oligonucleotides, which bind specifically to oncomiRs and inhibit their tumor‐promoting activity. A recent study reported that intranasal administration of cholesterol‐conjugated anti‐microRNA‐21 oligonucleotides effectively suppressed miRNA‐21 expression in Sprague Dawley rats [87]. MAPK1 was reported as a tumor promoter in GBM. By downregulating the expression of MAPK1 with miR‐362, the cell proliferation, migration, and invasion in GBM cells were inhibited in vitro [90]. The number of M2 macrophages is significantly elevated in glioma tissues and associated with tumor progression, and their abundance can be identified as a potential marker for tumor grade and prognosis [118, 119]. Within the glioma microenvironment, miR‐106b‐5p is downregulated in M1 macrophages but upregulated in M2 macrophages. This differential expression is mediated by miR‐106b‐5p targeting interferon regulatory factor 1, which promotes M1‐to‐M2 polarization. Therefore, inhibiting miR‐106b‐5p to block M2 polarization presents a potential therapeutic strategy for glioma treatment [91].

MiRNAs offer a unique therapeutic advantage due to their endogenous nature, small size, and ability to regulate multiple targets simultaneously through post‐transcriptional gene silencing. These features distinguish them from other RNA therapies and position miRNAs as versatile, efficient, and clinically promising tools for GBM treatment [120].

### 2.2 Peptides

Peptides are short‐chain amino acids, typically consisting of 2 to 50 residues linked by covalent peptide bonds. Their ease of synthesis and modification, low manufacturing cost, high diversity, and specificity make them attractive candidates for a wide range of therapeutic applications [128]. Peptides can be produced chemically or through biosynthesis in living cells, which corresponds to the classification of biological products despite their relatively small molecular weight. Over the years, peptides have been widely explored in the treatment of brain cancer with their intrinsic advantages in efficient cell penetration, tumor‐specific targeting, drug delivery platform, and direct therapeutic effects. Representative examples include receptor‐specific peptides such as internalizing arginine‐glycine‐aspartic acid (iRGD), and Angiopep‐2 for targeted delivery to brain tumors [129, 130, 131, 132, 133]; cell‐penetrating peptides (CPPs) like THR, LAH4, TAT, p28, which significantly enhanced transport across the BBB and enhanced intracellular uptake [134, 135, 136, 137]; and therapeutic peptides such as (KLAKLAK)_2_, known for inducing apoptosis specifically in cancer cells [138, 139] (Table 3). Among those peptides, CPPs gained particular attention as versatile and mechanistically distinct delivery vectors. CPPs are short peptides generally classified as cationic, amphipathic, or hydrophobic based on their physicochemical properties. They exhibit high efficiency and selectivity even at low concentrations [140]. Their cellular uptake is governed by peptide sequence, concentration, and membrane composition, and can occur through multiple pathways, including direct membrane penetration, adsorptive‐mediated transcytosis, and translocation through a transitory structure [141]. A major advantage of CPPs lies in their ability to covalently conjugate with macromolecular cargos, including proteins, DNA, and RNA, thereby overcoming the intrinsic impermeability of cellular membranes and enhancing both BBB penetration and intracellular delivery. Several CPPs also exhibit organelle‐specific targeting, further improving subcellular precision while reducing off‐target toxicity [140, 142].

**TABLE 3:** Summary of therapeutic peptides for treatment of brain cancer (organized by publication year).

Therapeutic agent	Carrier	Model/Tumor type	Therapeutic target	Administration	Key results	Year and Refs.
ICG‐p28 + TMZ	NA	In vitro in Mel‐7, MDA‐231BR, BCA‐1, A549 cell lines; in vivo in the MDA‐231BR‐bearing mice	p53‐p21 axis	IV	Enhanced accumulation in tumor lesions, improve the inhibitory effects of TMZ to tumor cells	2023 [153]
ICG‐p28 + TMZ	NA	In vitro in LN‐229 and U‐118 cell lines; in vivo in Athymic mice	p53/p21/CDK2 pathway	IV	Preferentially internalized by tumor cells, significantly enhance the anti‐tumor effect of TMZ	2022 [136]
AP1‐ELP‐KLAK	Elastin‐like polypeptide	In vitro in D54 and U87MG cell lines; in vivo in xenograft BALB/c nude mice	Caspase‐3 sensor	IV	Activated caspase‐3 and induced tumor apoptosis, exhibiting a 2∼3 fold increase in cell death compared to the control groups	2021 [139]

Abbreviations: IV, intravenous; FUC, focused ultrasound; IP, intraperitoneal; IC, intracranial; ICG, indocyanine green; AP1, interleukin‐4 receptor targeting peptide; ELP, elastin‐like polypeptide; NA, not applicable; TMZ, temozolomide.

Beyond therapeutic delivery, CPPs have demonstrated substantial potential in cancer diagnostics and image‐guided surgery. For example, the tumor‐targeting CPP p28 conjugated with indocyanine green (ICG‐p28) enabled high‐contrast near‐infrared visualization of tumor margins following systemic administration, facilitating precise discrimination between malignant and healthy tissues during surgical resection [143, 144]. CPPs have also been extensively engineered for multimodal imaging applications, including magnetic resonance imaging [145], positron emission tomography [146], and single‐photon emission computed tomography [147], through conjugation with fluorescent, metallic, or radioactive contrast agents. To further improve tumor specificity, activatable CPPs responsive to tumor‐associated stimuli, such as proteases, acidic pH, reactive oxygen species, or light, have been developed, providing controlled activation and enhanced imaging precision [148, 149, 150, 151]. These favorable characteristics have driven the development of numerous peptide‐based therapeutics extensively evaluated in preclinical studies. While chemically synthesized peptides for tumor targeting or enhanced penetration have been widely reviewed, this review focuses exclusively on biosynthetically produced peptides with intrinsic therapeutic properties. We critically evaluate their performance across preclinical brain cancer models and highlight their potential for clinical translation.

P28 is a 28‐amino‐acid CPP derived from the bacterial protein azurin. It functions as a multi‐target anticancer agent with the ability to selectively enter a variety of solid tumor cells. This selectivity is likely mediated via caveolae‐dependent endocytosis, which is more active in cancer cells, enhancing tumor‐specific targeting. Upon entry, P28 binds to and stabilizes the tumor suppressor protein p53 by inhibiting its ubiquitination and degradation, leading to G2/M cell cycle arrest and tumor cell death [152]. The combined tumor‐targeting and growth‐inhibitory properties make p28 a promising therapeutic agent. Mander and colleagues investigated ICG‐p28 for image‐guided surgery and therapy [136]. In vivo studies showed that ICG‐p28 was preferentially taken up and retained at tumor site, providing a clear contrast between tumor cells and normal brain tissue. Furthermore, coadministration of p28 with DNA‐damaging drugs such as temozolomide enhanced cytotoxicity in GBM‐bearing mice [136]. The same research group further evaluated their formulation in a brain metastases mouse model with an intact BBB. Intravenously administered ICG‐p28 was confirmed to penetrate the BBB and specifically accumulate in brain tumor lesions, remarkably enhancing temozolomide‐induced apoptosis via modulating the p53‐p21 axis [153]. Inspired by the preclinical success, p28 has progressed to clinical trials, demonstrating promising outcomes. A phase I clinical trial involving 18 children with recurrent or progressive CNS tumors (NCT01975116) reported good tolerability and limited toxicity. While p28 alone may have limited efficacy, combination strategies with other chemotherapeutic agents may enhance therapeutic outcomes [154].

(KLAKLAK)_2_ (KLAK) is a pro‐apoptotic peptide with an alpha‐helical structure that allows interaction with cellular membranes. Once internalized, KLAK binds to the negatively charged outer mitochondrial membrane, triggering mitochondrial permeabilization. This process increases reactive oxygen species, disrupts mitochondrial function, and induces mitochondria‐dependent apoptosis [138]. However, KLAK suffers from limited stability and poor cancer cell specificity, which can cause side effects and limit its therapeutic potential. To address these limitations, targeting ligands and delivery platforms have been developed. Given the robust expression of interleukin‐4 receptor (IL‐4R) on cancer cells and its role in metastasis and apoptosis resistance, an IL‐4R‐targeting peptide significantly enhanced KLAK's tumor specificity [139]. KLAK‐containing elastin‐like polypeptides (ELP) were synthesized by transforming ELP‐KLAK and AP1‐ELP‐KLAK genes into BL21 *E. coli* using a pET 25b^+^ vector. Conjugation with IL‐4R enabled intravenous delivery in D54/effluc tumor‐bearing mice, inducing apoptosis, increasing caspase‐3 sensor activity in a time‐dependent manner, and significantly inhibiting tumor growth [139].

Beyond delivery and cytotoxic peptides, peptide‐based vaccines have also progressed into clinical evaluation for brain cancer immunotherapy [155]. These vaccines aim to induce tumor‐specific immune responses by targeting tumor‐associated antigens. One of the most studied approaches is the EGFRvIII‐targeted vaccine rindopepimut, which targets a mutation found only in tumor cells and not in normal brain tissue [156]. This vaccine was evaluated in Phase II clinical trials (NCT00458601) and a large Phase III study (NCT01480479) in glioblastoma patients [157]. Although early trials showed that it could activate immune responses against tumor cells, the Phase III trial did not improve overall survival, largely due to tumor heterogeneity and the loss of the EGFRvIII target during disease progression [157, 158].

Another strategy focuses on vaccines targeting specific genetic mutations inside tumor cells. For example, IDH1‐R132H peptide vaccines target a common mutation found in lower‐grade gliomas. This mutation is stable and tumor‐specific, making it an attractive vaccine target. Early clinical evaluation (NCT02454634) has shown that this vaccine can safely activate immune responses in patients, with no major toxicity reported [159, 160]. In addition, personalized neoantigen peptide vaccines are being developed based on each patient's individual tumor mutations. These vaccines are designed by identifying unique tumor‐specific genetic alterations and using them to create customized peptides that train the immune system to recognize the tumor. Early clinical studies (NCT02287428, NCT06314087) have demonstrated that this approach can induce strong and highly specific T‐cell immune responses in glioma patients [161].

Overall, these clinical studies highlight that while peptide vaccines can successfully stimulate anti‐tumor immunity in brain cancer, their effectiveness is still limited by tumor evolution and immune escape mechanisms (Table 4). Collectively, peptide‐based therapeutics for brain cancer span targeting peptides, cell‐penetrating sequences, cytotoxic effectors, and vaccine platforms, demonstrating strong versatility across drug delivery and immunotherapy applications. Preclinical studies consistently show that peptides can enhance BBB penetration, improve tumor selectivity, and enable multifunctional therapeutic design when integrated with carriers or targeting ligands. However, clinical translation remains limited, with only a small number of peptide‐based strategies advancing into human trials and even fewer demonstrating clear survival benefit. Key challenges include rapid enzymatic degradation, insufficient tumor specificity in heterogeneous gliomas, immune escape mechanisms, and variability in delivery across the BBB. Therefore, future progress will depend on rational peptide engineering, improved stabilization strategies, and combination with advanced delivery systems and predictive human‐relevant models to bridge the gap between promising preclinical performance and effective clinical translation.

**TABLE 4:** Summary of clinical trials conducted using peptides for treatment of brain cancer (organized by completion year).

Therapeutic agent	Category	Delivery approach	Application/indication	Status	NCT number
IDH 1 peptide vaccine	Peptide	Subcutaneous injection	IDH1R132H‐mutated, grade III‐IV gliomas	Completed 2017	NCT02454634 (phase I)
Rindopepimut (CDX‐110) + Temozolomide	Peptide	Intradermal injection	GBM multiforme	Completed 2016	NCT00458601 (phase II)
Rindopepimut (CDX‐110) + Temozolomide	Peptide	Intradermal injection	Newly diagnosed GBM	Completed 2016	NCT01480479 (phase III)
P28	Peptide	Intravenous injection	DMG, High‐grade glioma, Medulloblastoma, Pineoblastoma, etc.	Completed 2015	NCT01975116 (phase I)
Multi‐peptide (tumor‐associated antigens) + Montanide ISA‐51VG	Peptide Vaccine	Subcutaneous injection	Recurrent or Refractory CNS Tumors	Completed 2014	NCT00935545 (phase I)
Gp96 heat shock protein‐peptide complex	Peptide Vaccine	Intradermal injection	Recurrent or progressive high‐grade glioma	Completed 2013	NCT00293423 (phase I/II)
Dendritic cells + glioma‐associated antigen peptide	Peptide Vaccine	Intradermal injection	Malignant glioma	Completed 2012	NCT00612001 (phase I)
P28	Peptide	Intravenous injection	Refractory Solid Tumors	Completed 2011	NCT00914914 (phase I)
Peptide vaccine + Radiotherapy	Peptide	NA	Refractory Solid Tumors	Recruiting	NCT06314087 (phase II)
NeoVax Vaccine + Pembrolizumab + Temozolomide	Peptide	NA	Newly diagnosed GBM	Recruiting	NCT02287428 (phase I)

Abbreviations: CNS, central nervous system; DMG, diffuse midline glioma; GBM, glioblastoma; NA, not applicable.

### 2.3 Proteins

Proteins are large, complex molecules composed of amino acids, the fundamental building blocks for cells and tissues in all living organisms. Their unique amino acid sequences and three‐dimensional structures confer remarkable functional diversity, enabling a wide range of applications in brain cancer research and therapy. In this context, proteins can serve as targeting ligands for tumor‐specific markers, drug delivery platforms, or reliable biomarkers for early diagnosis and treatment monitoring. Representative examples include monoclonal antibodies (e.g., Bevacizumab, Cetuximab) for selective cancer targeting; receptor‐specific ligands (e.g., lactoferrin, transferrin) facilitating BBB transcytosis; protein‐based drug delivery systems (e.g., Ferritin, Albumin), and tumor‐associated proteins such as glial fibrillary acidic protein used for diagnostic purposes. This part focuses on proteins with therapeutic potential in brain cancer.

Antibodies are specialized proteins produced by the immune system in response to foreign antigens. Monoclonal antibodies have become a leading class of therapeutics, with over 50 products approved for clinical use and more than 1800 mAb‐based therapies currently in the clinical stage [22]. Their growing success is largely attributed to their high target specificity and favorable pharmacokinetics compared to small‐molecule drugs. To date, bevacizumab is the only protein‐based drug approved by the Food and Drug Administration for the clinical treatment of brain cancer. Other protein drugs, such as the programmed cell death protein 1 (PD‐1) immune checkpoint inhibitors Nivolumab (Opdivo), Pembrolizumab (Keytruda), and Atezolizumab (Tecentriq), as well as the anti‐CTLA‐4 checkpoint inhibitor Ipilimumab (YERVOY) and Cetuximab (Erbitux), an epidermal growth factor receptor (EGFR) targeting monoclonal antibody, are not primarily used for brain cancer treatment. Besides BBB‐mediated restriction, brain tumors, particularly GBM, are often described as “cold tumors” due to their profoundly immunosuppressive microenvironment [162]. This environment limits immune cell infiltration and results in low levels of cytotoxic T lymphocytes, limiting the effect of PD‐1 blockade [163, 164]. However, these agents have shown potential and are under investigation in preclinical studies and clinical trials for their applicability in brain cancer or brain metastases therapy, as summarized in Table 5.

**TABLE 5:** Summary of therapeutic proteins for the treatment of brain cancer (organized by publication year).

Therapeutic agent	Carrier	Model/Tumor type	Therapeutic target	Administration	Key results	Year and Refs.
CTX, Dox	Exosome	In vitro in C6 cell line; in vivo in xenograft mouse model	EGFR	IV	CTX‐Exo‐DOX prolonged survival 1.47‐fold compared to the DOX group	2024 [123]
Cetuximab, capecitabine, oxaliplatin	NA	In a colorectal cancer patient with brain metastasis	EGFR	IV	Two weeks of treatment significantly reduced the right cerebellar mass and resolved surrounding edema.	2023 [186]
Cetuximab, capecitabine, oxaliplatin	NA	In a colorectal cancer patient with brain metastasis	EGFR	IV	Two weeks of treatment significantly reduced the right cerebellar mass and resolved surrounding edema.	2023 [186]
^89^Zr‐CTX	NA	In vitro in U251 (EGFR^+^) cell line; in vivo in a xenograft mouse model	EGFR	FUC + µB + IV	No improvement in CTX long‐term accumulation, retention in tumors, or related mice survival with FUS	2023 [176]
aPD‐L1 Antibody	NA	In vivo in Porcine Model	aPD‐L1	FUS + microbubbles	Significantly increased anti‐PD‐L1 antibody delivery volume and accumulation in the brain	2023 [187]
mCD47	NA	In vitro and in vivo in GL261 glioma cell line and C57BL/6 mice	CD47–signal regulatory protein‐α axis	FUS	Effectively controlled tumor growth and prolonged survival in mice	2021 [188]
Protein	Exosomes	In vitro in hCMEC/D3 and C17.2 cell lines	NA	NA	C17.2 neural stem cell exosomes efficiently delivered proteins across an in vitro BBB model by receptor‐mediated endocytosis	2021 [124]
Anti‐PD‐1, EGFRvIII CAR T cells, CXCL10‐secreting APCs	NA	In vitro in GL261 and U87 cell lines; in vivo in C57BL/6 and NSG mice	PD‐1, EGFRvIII,	LIPU + IV	Combining anti‐PD‐1 with LIPU increased its localized accumulation in the brain parenchyma; CAR T‐cell with LIPU increased median survival by greater than 129%	2021 [189]
Monoclonal antibody	NA	In vivo in SCID/NCr mice	NA	IA/ IV	IA delivery achieved faster, higher cerebral uptake than IV injection	2020 [190]
GC118, CTX	NA	In vitro in GBM patient‐derived tumor cells; in vivo in Xenograft BALB/c nude model	EGFR	IP	Induced apoptosis and inhibited tumor cell growth, thereby improving median overall survival	2020 [191]
CTX–IRDye800	NA	In vivo in GBM39 patient‐derived cells and Xenograft Nu/nu mouse model	EGFR	IP	6.2‐fold cerebral NIR‐I fluorescence enhancement in treated models; offering visualized image‐guided surgical removal	2020 [178]
CTX, CHC	PLGA &. chitosan	In vitro in the U251 and SW1088 cell lines	EGFR	NA	Exhibited a greater inhibition effect than the isolated drugs	2020 [126]
Bevacizumab	PLGA nanoparticles	In vitro in J774A.1 cell line; in vivo in CD‐1, C57BL/6, and orthotopic nude mice	VEGF	IN	Improved cerebral penetration and prolonged residence time of bevacizumab	2019 [127]
Bevacizumab	NA	In patient with malignant GBM	VEGF	IA	MRI‐guided IA bevacizumab infusion rapidly improved neurological status and reduced tumor volume	2019 [192]
Nimotuzumab, Trastuzumab	Nanocapsules	In vitro in U87‐EGFRwt and MDA‐MB‐231/HER2 cell lines; in vivo in the BALB/c mouse model	EGFR, HER2 receptor	IV	Inhibited tumor growth and prolonged mouse survival time	2019 [193]
Isotype IgG, Anti‐TfR^A^, Anti‐TfR^D^, Anti‐TfR^A^/BACE1	Gold nanoparticle	In vitro in the BCECs cell line; in vivo in the C57BL/6 mouse model	Transferrin receptor	IV	Monovalent (bi‐specific) antibody modification showed better uptake capacity compared to high‐ and low‐affinity antibodies	2018 [194]

Abbreviations: IV, intravenous; FUC, focused ultrasound; IP, intraperitoneal; IC, intracranial; IA, intraarterial; LIPU, low‐intensity pulsed ultrasound; CAR, chimeric antigen receptor; EGFRvIII, epidermal growth factor receptor variant III; VEGF, vascular endothelial growth factor; CTX, cetuximab; Dox, doxorubicin; EGFR, epidermal growth factor receptor; TFR, transferrin receptor; HER2, human epidermal growth factor 2 receptor; CHC, alpha‐cyano‐4‐hydroxycinnamic acid; PLGA, poly(lactic‐co‐glycolic acid); aPD‐L1, anti‐programmed cell death‐ligand 1; NA, not applicable; hCMEC/D3, human cerebral microvascular endothelial cell.

Bevacizumab has been widely investigated in anti‐tumor therapy for its anti‐angiogenesis effect and is clinically used for the treatment of brain cancers such as GBM. By binding to vascular endothelial growth factor (VEGF), bevacizumab forms an inactive complex that prevents VEGF from interacting with its endothelial receptors, thereby inhibiting the formation of new blood vessels. Although VEGF is produced by normal cells, tumor cells often overexpress this growth factor due to their highly proliferative and infiltrative nature. The excessive VEGF secretion promotes angiogenesis, supplying nutrients and oxygen that support tumor growth beyond a limited size. Bevacizumab selectively neutralizes VEGF secreted primarily from tumor cells, effectively restricting tumor vascularization and growth. Bevacizumab gained approval from the FDA for the treatment of Metastatic Colorectal Cancer in 2004 as the first anti‐VEGF drug. It was later approved as a first‐line treatment for several other malignancies, including GBM, in 2017. However, it has not been approved for brain tumors in the United Kingdom, largely due to a lack of significant improvement in overall patient survival [165].

Although Bevacizumab has been extensively used for GBM treatment, its clinical benefit remains limited. Large‐scale studies have failed to demonstrate a significant improvement in overall survival in GBM patients receiving bevacizumab [166, 167]. Combination therapy with Bev and Temozolomide has shown modest improvements in progression‐free survival and overall response rates, but no benefits in overall survival [168, 169]. Nevertheless, bevacizumab can improve patient quality of life by reducing tumor vascular permeability and peritumoral oedema, which alleviates neurological symptoms and cognitive impairment [168, 170]. A major limitation of bevacizumab therapy is the rapid development of resistance. GBM can activate alternative angiogenic pathways to sustain its blood supply despite VEGF inhibition. Moreover, VEGF blockade may induce hypoxia within the tumor microenvironment, triggering adaptive survival mechanisms that promote a more invasive tumor phenotype. Limited penetration across the BBB also restricts the therapeutic efficacy of bevacizumab. Due to its large molecular weight (∼149 kDa), intravenously administered bevacizumab exhibits poor BBB permeability, resulting in insufficient drug accumulation in tumor tissue [171].

To overcome this limitation, strategies to transiently disrupt the BBB have been explored. Osmotic blood–brain barrier opening (OBBBO) combined with intra‐arterial drug delivery has shown promise in enhancing Bev transport to the brain. Preclinical studies demonstrated that OBBBO followed by intra‐arterial administration remarkably increased the cerebral and tumor uptake of radiolabeled bevacizumab in mice, whereas intravenous infusion did not [172]. Consistently, a clinical study in 70 patients demonstrated the safety and feasibility of intra‐arterial cerebral infusion of bevacizumab or Cetuximab following osmotic BBB disruption [173]. In the bevacizumab‐treated cohort, preliminary data showed a median overall survival of 24 months and a post‐recurrence survival of 13.1 months. These outcomes compare favorably with prior trials using intravenous bevacizumab, which have shown no significant benefit in overall survival in 10 studies (*n* = 2515) [174]. These findings highlight the potential of BBB‐disruption strategies to enhance the therapeutic efficacy of biologics in brain tumors, while underscoring the BBB as a major barrier to treatment. However, improving BBB transport alone is insufficient because GBM exhibits substantial tumor heterogeneity and requires patient‐specific therapeutic stratification. For example, an in silico analysis of 1,082 glioma patients showed that approximately 20% of primary GBMs lacked VEGFA expression, suggesting limited responsiveness to bevacizumab [175]. This was further supported by both in vitro and in vivo GBM models. Although bevacizumab did not improve overall survival in the full cohort, patients with high VEGFA expression showed a median overall survival benefit of 8.5 months. In contrast, no survival benefit was observed in patients with low VEGFA expression [175].

Another well‐studied monoclonal antibody for cancer treatment is Cetuximab (CTX), a human‐murine chimeric antibody that selectively binds to the EGFR receptor on cancer cell surfaces. By blocking receptor‐associated kinases activation, CTX inhibits the growth and survival of epidermal growth factor receptor (EGFR)‐expressing tumor cells. It has been approved, either as a monotherapy or in combination with chemotherapy or radiotherapy, for the treatment of squamous cell carcinoma and colorectal cancer, where it significantly improves overall survival (OS) and objective response rate [176]. However, clinical trials in GBM have failed to reproduce comparable efficacy, despite the frequent overexpression of EGFR in these tumors [177]. The limited therapeutic response is largely attributed to the restrictive BBB and the heterogeneous blood‐tumor barrier, which hinders antibody penetration to the tumor site. To overcome this, a natural exosome‐based delivery platform, CTX‐Exo‐DOX, was developed to co‐deliver CTX and DOX to GBM [123]. This system enhanced BBB penetration and increased CTX accumulation in glioma cells in vitro. Intravenous administration in C6 tumor‐bearing mice significantly suppressed tumor growth and promoted apoptosis [123]. Beyond its direct therapeutic role, the high tumor specificity of CTX has also been exploited for targeted applications. CTX conjugated with the fluorescent dye IRDye800 has been utilized to distinguish malignant tissue from healthy brain parenchyma, facilitating fluorescence‐guided resection and ex vivo pathological validation [178]. In addition, CTX functionalized with alpha‐cyano‐4‐hydroxycinnamic acid–loaded Poly(lactic‐co‐glycolic acid) (PLGA) nanoparticles achieved superior tumor inhibition compared with the administration of the free drug [126].

Given the promising preclinical outcomes, several monoclonal antibodies have progressed to clinical evaluation, including nivolumab (NCT02460068, NCT02320058), trastuzumab (NCT03714243), bevacizumab (NCT01884740, NCT01884740), rituximab (NCT00068250), atezolizumab (NCT03526900) and omburtamab (NCT01502917) as summarized in Table 6. In a phase I clinical trial (NCT01884740), bevacizumab and cetuximab were co‐delivered intra‐arterially following mannitol‐induced BBB disruption in patients with DMG and GBM [179]. Six of ten DMG patients showed clinical improvement, with 17.3 months mean overall survival [179]. Trastuzumab, although not primarily approved for brain tumors, was safely and efficiently delivered across the BBB using magnetic resonance imaging (MRI)‐guided focused ultrasound in four HER2‐positive patients with progressive brain metastases (NCT03714243). Their results revealed enhanced antibody accumulation and therapeutic activity at targeted sites [180]. Similarly, nivolumab combined with ipilimumab demonstrated durable efficacy in melanoma patients with asymptomatic brain metastases, as sustained responses were observed over a seven‐year follow‐up phase III NIBIT‐M2 trial [181].

**TABLE 6:** Summary of clinical trials conducted using proteins for treatment of brain cancer (organized by completion year).

Therapeutic agent	Category	Delivery approach	Application/indication	Status	NCT number
Atezolizumab, Temozolomide	Antibody	IV	GBM	Completed 2025	NCT03174197 (phase I/II)
Nivolumab, Bevacizumab	Antibody	IV	GBM	Completed 2024	NCT03890952 (phase II)
Lerapolturev + Pembrolizumab	Antibody	IV	Recurrent GBM	Completed 2024	NCT04479241 (phase II)
D2C7	Antibody, Immunotoxin	CED	Recurrent glioma	Completed 2024	NCT02303678 (phase I)
Atezolizumab, Carboplatin, Pemetrexed	Antibody	IV	Advanced nonsmall cell lung cancer with brain metastasis	Completed 2022	NCT03526900 (phase II)
GC1118	Antibody	IV	Recurrent GBM	Completed 2022	NCT03618667 (phase II)
Trastuzumab	Antibody	IV+ FUS+ Nuclear localizing signal peptides	Her2‐positive breast cancer brain metastasis	Completed 2022	NCT03714243 (phase I)
[^124^I]‐Omburtamab	Antibody	CED	DMG	Completed 2022	NCT01502917 (phase I)
Human‐ Recombinant Bone Morphogenetic Protein 4	Protein	CED	Progressive and/or multiple recurrent Glioblastoma multiforme	Completed 2021	NCT02869243 (phase I)
Nivolumab, Ipilimumab	Antibody	IV	Melanoma brain metastases	Completed 2020	NCT02320058 (phase II)
Pembrolizumab	Antibody	IV	Non‐small cell lung cancer or melanoma with brain metastasis	Completed 2020	NCT02085070 (phase II)
MDNA55	Protein	CED	Recurrent or Progressive GBM	Completed 2019	NCT02858895 (phase II)
Rituximab, Temozolomide, Methotrexate	Antibody	IV	CNS lymphoma	Completed 2016	NCT00068250 (phase I/II)
IL13‐PE38QQR	Protein	CED	First recurrence of GBM multiforme	Completed 2007	NCT00076986 (phase I)
Nivolumab, Temozolomide, Relatlimab, Radiation	Protein	IV	IDH wildtype newly diagnosed GBM	Recruiting	NCT06816927 (phase II)
Cetuximab, Bevacizumab	Antibody	IA	Glioma	Recruiting	NCT05956821 (phase I/II)
2141‐V11, D2C7‐IT	Antibody, Immunotoxin	CED	Recurrent glioma	Recruiting	NCT04547777 (phase I)
Pembrolizumab	Antibody	IV	Brain Metastasis	Active, not recruiting	NCT02886585 (phase II)
OS2966	Antibody	CED	High‐grade Glioma	Terminated	NCT04608812 (phase I)
Cetuximab‐IRDye800	Antibody	IV	GBM, Diffuse astrocytoma	Terminated	NCT02855086 (phase I)
Bevacizumab, Cetuximab	Antibody	IA	DMG, GBM	Terminated	NCT01884740 (phase I)
OS2966 + Gadoteridol	Antibody	CED	High‐grade Glioma	Terminated	NCT04608812
Omburtamab	Antibody	CED	DMG	Withdrawn	NCT05063357
Nivolumab, Ipilimumab	Antibody	IV	Melanoma Brain Metastasis	Unknown	NCT02460068 (phase III)
Cetuximab	Antibody	IA	Recurrent malignant glioma	NA	[195] (phase I)

Abbreviations: IV, intravenous injection; CED, convection‐enhanced delivery; CNS, central nervous system; DMG, diffuse midline glioma; GBM, glioblastoma; IDH, isocitrate dehydrogenase; NA, not applicable.

Despite the success of immune checkpoint inhibitors in cancers such as melanoma and brain metastases, their efficacy in GBM remains limited. In randomized trials, adjuvant anti‐PD‐1 therapy (e.g., nivolumab) combination with radiotherapy and temozolomide after maximal resection showed no survival benefit over the standard Stupp protocol [182, 183, 184]. This may reflect the highly immunosuppressive tumor microenvironment, limited tumor‐infiltrating lymphocytes, and restricted macromolecular drug access to the brain parenchyma [185]. Recent evidence suggests that combination immune checkpoint blockade may overcome these limitations. A triplet neoadjuvant regimen of nivolumab (anti‐PD‐1), ipilimumab (anti‐cytotoxic T‐lymphocyte protein 4), and relatlimab (anti‐lymphocyte‐activation gene 3) in a newly diagnosed IDH‐wild‐type GBM patient increased the diversity and activation of tumor‐infiltrating lymphocytes after treatment [185]. The patient remained recurrence‐free at 17 months post‐therapy, highlighting the potential of neoadjuvant combination immune checkpoint inhibitors to enhance T‐cell infiltration and activation. To further evaluate this approach, the GIANT trial (NCT06816927) is ongoing.

Antibody‐based therapies, particularly bevacizumab, exemplify the promise of biologics in targeting critical molecular pathways that regulate angiogenesis and tumor growth in brain cancers. Other monoclonal antibodies (e.g., cetuximab, omburtamab, and trastuzumab), though not primarily approved for brain tumors, have also shown therapeutic potential in treating brain metastases and gliomas under clinical scenarios when combined with innovative delivery approaches. Despite these encouraging findings, the overall survival benefit of antibody therapies remains limited, mainly due to restricted BBB permeability, tumor heterogeneity, and adaptive resistance mechanisms. Clinical studies employing techniques such as mannitol‐induced BBB disruption, MRI‐guided focused ultrasound, and convection‐enhanced delivery have demonstrated improved antibody penetration and localized delivery. Continued advancements in antibody engineering, nanocarrier systems, and real‐time imaging–guided delivery are expected to further enhance brain accessibility and therapeutic performance, paving the way for more effective biologic interventions against brain cancer.

### 2.4 Cell Therapy

Live‐cell therapy is an innovative approach that utilizes living cells to regulate or facilitate critical biological processes for disease treatment, including cancer. Among these, reengineered immune cells such as Chimeric antigen receptor (CAR) T cells, macrophages, and natural killer (NK) cells have demonstrated promising therapeutic potential in oncology [196]. CAR T‐cell therapy which collects and modifies patient‐derived T cells to recognize and eliminate tumor cells. It remains the most extensively studied and potent approach but is limited by autologous dependence and neurotoxicity risks. To address the need for timely intervention in rapidly progressing tumors, allogeneic CAR macrophages and CAR NK cells have been developed, offering “off‐the‐shelf” immune effectors without patient‐specific engineering (Figure 3). CAR macrophages enhance phagocytic activity and remodel the tumor microenvironment, whereas CAR NK cells exhibit favorable safety profile but shorter persistence. By leveraging both innate and adaptive immune mechanisms, these cell‐based therapies offer a personalized approach with the potential for broad applicability in cancer treatment (Table 7).

**FIGURE 3:**
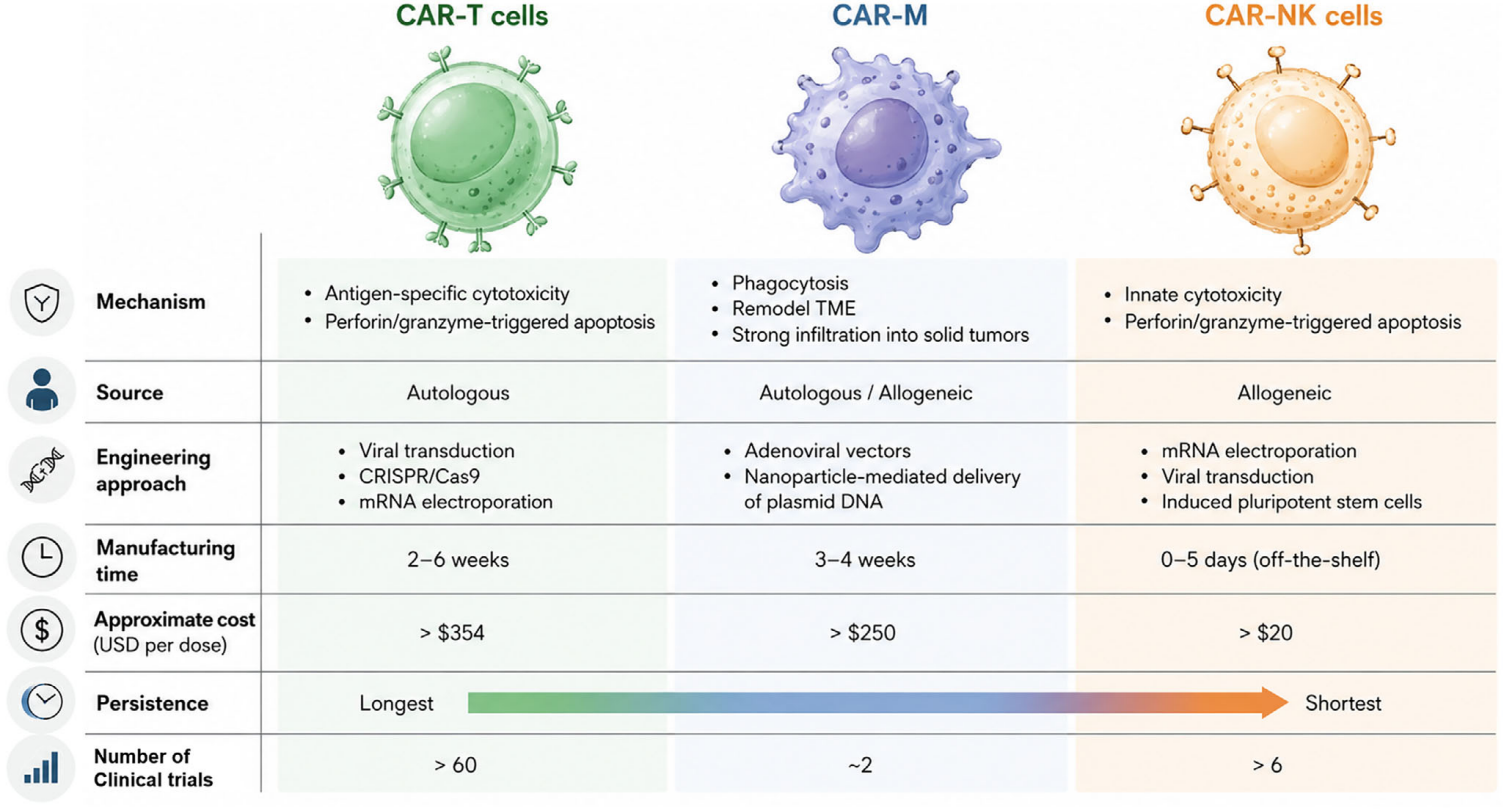
Comparison of CAR‐T cell, CAR natural killer (CAR‐NK) cell, and CAR‐macrophage (CAR‐M) therapies for brain cancer. CAR T cells remain the most extensively studied platform, with numerous ongoing clinical trials in brain cancer. A key advantage is their ability to form memory T cells, enabling long‐term persistence and durable antitumor responses. In contrast, CAR‐NK cells generally exhibit shorter in vivo persistence due to their limited lifespan and lack of sustained memory formation [237]. Manufacturing timelines also differ across platforms. Autologous CAR T‐cell and CAR‐M therapies typically require cell expansion or differentiation ex vivo, followed by processing, quality control, and shipment, resulting in vein‐to‐vein times of approximately 3–4 weeks or longer [276]. By comparison, CAR‐NK cells can be produced as allogeneic, off‐the‐shelf products, enabling more rapid availability, which is particularly advantageous for patients with aggressive brain tumors [238]. Approximate costs are expressed in millions of USD.

**TABLE 7:** Summary of therapeutic cells for the treatment of brain cancer (organized by publication year).

Therapeutic agent	Carrier	Model/ Tumor type	Therapeutic target	Administration	Key results	Year and Refs.
GD2‐CAR T cell	NA	In vitro in DMG, pHGG, and H3 mutant cell lines; In vivo in NOD‐SCID‐γc–/– mice	GD2	IT	mRNA‐transfected GD2‐directed CAR T cells effectively targeted DMG and high‐grade glioma without inducing neurotoxicity	2025 [229]
CAR‐encoding plasmid	Mesenchymal stem cells	In vitro in RAW 264.7, Luci^+^ GL261, 3T3‐L1, Luci^+^ CT2A cell line, and in murine adipose‐derived mesenchymal stem cells; In vivo in C57BL/6 mice	Glioma‐associated microglia/macrophages	IC, IV	In vivo generation of CAR‐macrophages Elevated cytotoxic T cells and M1‐like macrophages within tumors contribute to antitumor effects	2025 [125]
Plasmid DNA	Nanocomplexes	In vitro and in vivo in BMDM, GL261, 293 T, and RAW264.7 cell lines; in vivo in C57BL/6 mice	M2 tumor‐associated macrophages	CED	Reprogrammed intratumoral macrophages with tumor‐specific CARs, restoring their innate anticancer activity within the TME in vivo	2023 [121]
CAR‐T cell, CXCL11‐grafted oncolytic adenovirus	NA	In vitro in U251, A172, U87, T98G, and GL261 GBM cell lines, and 293A, 293T, A549, H4 cell lines; in vivo in NCG and C57BL/6 mice	B7H3	IV, IT	CXCL11‐modified oncolytic adenoviruses synergize with CAR T‐cells to enhance immune‐virotherapy	2023 [204]
EGFRvIII‐CAR‐T cell, anti‐VEGF antibody B20	NA	In vitro in CT2A and GSC005 GBM cell lines; in vivo in C57BL/6 mice	EGFRvIII	IV	Anti‐VEGF therapy promoting EGFRvIII‐CAR T cells infiltration and distribution within the TME	2023 [205]
Anti‐CD133 CAR plasmid, CD47 antibody	Nanoporter‐hydrogel	In vitro in RAW 264.7, THP‐1, BMDM, 293T, or GL261 cell lines; in vivo in GL261‐bearing mice	Macrophages and microglia	Intracavity injection	Achieved the in‐situ generation of CAR‐macrophages Effectively suppressed postsurgical GBM relapse	2022 [202]
EGFR‐CAR NK cells, Oncolytic viral	NA	In vitro in 293T cell line and CT2A, U251, LN229, U87vIII GBM cell lines; in vivo in NOD/SCID/IL‐2rg mice	EGFR	IC	Enhanced intracranial infiltration and elevated CAR NK cell levels, with extended mouse survival	2021 [230]
CAR‐interferon‐γ‐encoding plasmid DNA	Nanocomplexes	In vitro in Neuro‐2a, RAW264.7, L929 cell lines; in vivo in A/J mice	M2 tumor‐associated macrophages	IV	Programming M2 tumor‐associated macrophages in the TME in vivo to an anti‐tumor M1 phenotype expressing CAR to eliminate cancer cells	2021 [122]
NK cells	NA	In vitro in C6 GBM cell line	HSP70	IC	Activated NK cells successfully crossed the BBB and targeted GBM tumor cells	2020 [231]
Bispecific T cell engager secreted macrophages	NA	In vitro in U87 MG, 293T cell line; in vivo in NSG mice	EGFRvIII	IC	Enhanced T cell activation and effectively inhibited tumor growth both in vitro and in vivo	2020 [232]
HER2‐CAR‐T cell, anti‐PD1 antibody	NA	In vitro in U251, U87 GBM cell lines and 293T‐17 cell line	HER2	NA	Anti‐PD‐1 blockade enhanced the efficacy of HER2‐CAR T cells against U251 GBM cells	2019 [233]
CD4^+^/ CD8^+^ CAR‐T cell	NA	In vitro in PBT030‐2 and PBT103‐2 patient‐derived GBM cell lines; in vivo in NOD/SCID/IL2R^–/–^ mice	IL13Rα2	IC	CD8^+^ CAR T cells exhibited potent, transient responses, while CD4^+^ CAR T cells provided sustained antitumor activity	2018 [234]
CAR‐KHYG‐1 NK cells	NA	In vitro in U87 MG, KHYG1, and HEK293T cell lines	EGFRvIII	NA	EGFRvIII‐specific‐CAR‐nature killer cells inhibited GBM growth via apoptosis in vitro	2018 [235]

Abbreviation: IT, intratumoral; IC, intracranial; TME, tumor microenvironment; EGFRvIII, epidermal growth factor receptor variant III; IL13Rα2, IL‐13 receptor α2; NA, not applicable.

#### 2.4.1 CAR‐T Cell Therapy

CAR T cell therapy represents the forefront of autologous immunotherapy to overcome tumor immune evasion. While native T cells rely on major histocompatibility molecules (MHC)‐mediated antigen presentation, tumors frequently escape immune surveillance by downregulating MHC expression [197, 198]. Genetically engineering patient‐derived T‐cells to express chimeric antigen receptors (CARs) circumvents this limitation, enabling direct, antigen‐specific tumor elimination independent of MHC [122, 199, 200, 201, 202]. Following reinfusion, CAR T‐cells can expand and persist, providing durable and personalized antitumor activity. However, the application of CAR T‐cell therapy in GBM and other malignant brain tumors remains challenging. Key barriers include an immunosuppressive microenvironment, tumor heterogeneity, restricted trafficking across the BBB, and treatment‐related toxicities toxicity [196, 199, 203].

To address these limitations, combinatorial strategies have been explored. For example, CXCL11‐grafted oncolytic adenovirus was found to reprogram the immunosuppressive TME and increase the infiltration of CAR‐T cells to achieve a durable antitumor response [204]. Anti‐VEGF therapy has been shown to normalize tumor vasculature and improve the EGFRvIII‐directed CAR T cells intratumoral distribution [205]. Traditional CAR T‐cell manufacturing relies on viral vectors or transposon‐based gene integration, is constrained by complex manufacturing, high cost, inconsistent integration efficiency, and limited long‐term cell persistence in solid tumors [206, 207]. To address these issues, lipid nanoparticles were used to deliver CAR‐encoding mRNA directly to T cells, enabling rapid and efficient CAR expression in vivo [207, 208]. This approach prolonged CAR activity while enabling rapid and cost‐effective manufacturing. To further enhance translation effectiveness, 24 ionizable lipid nanoparticles were synthesized and screened [209]. The lead formulation achieved CAR expression comparable to electroporation but with substantially lower cytotoxicity and potent antitumor activity.

While CAR‐T cell therapies have gained remarkable success in various hematologic malignancies, their clinical application remains in the early stages for brain cancer. Among the 42 FDA‐approved cell and gene therapies, CAR‐T cell therapy accounts for 17.1%, second only to umbilical cord blood derivatives, representing 9 (22.0%) of 41 therapies. The first approved CAR‐T cell therapy was Tisagenlecleucel in 2017, for an 82.5% overall remission rate among 63 patients in a phase II trial [210]. Following, several CAR‐T cells therapies gained approval, including idecabtagene vicleucel (Abcema) and ciltacabtagene autoleucel (Carvykti) are used for treating multiple myeloma; obecabtagene autoleucel (Aucatzyl), lisocabtagene maraleucel (Breyanzi), tisagenlecleucel (Kymriah), brexucabtagene autoleucel (Tecartus) and axicabtagene ciloleucel (Yescarta), which are used for B‐cell lymphoma. These achievements have accelerated the clinical translation of CAR T cell therapy for brain tumors. Recently, a Phase I clinical trial (NCT04185038) evaluating intracranial delivery of B7‐H3‐targeted CAR T cells in DMG reported a median overall survival of 19.8 months, markedly exceeding the historical median of 11.2 months [211]. This completed first‐in‐human study encouraged an increasing number of clinical trials for brain tumors, targeting diverse tumor‐associated antigens (e.g., CSPG4, HER2, IL‐13Rα2, GD2, B7‐H3, and EGFRvIII as shown in Table 8).

**TABLE 8:** Summary of clinical trials conducted using cell therapies for treatment of brain cancer (organized by completion year).

Therapeutic agent	Category	Delivery approach	Application/indication	Status	NCT number
EGFRvIII T cells + Pembrolizumab	CAR‐T Cells	IV	Newly diagnosed, EGFRvIII^+^, MGMT‐unmethylated glioblastoma	Completed 2021	NCT03726515 (phase I)
ALECSAT cell + TMZ + Radiation	NK cells, Cytotoxic T cells	IV	Newly Diagnosed GBM	Completed 2020	NCT02799238 (phase II)
Autologuos Lymphoid Effector Cells + TMZ + Radiation	Lymphoid effector cells	NA	Newly diagnosed GBM	Completed 2020	NCT02799238 (phase II)
Anti‐EGFRvIII CAR T cells	CAR‐T Cells	IV	Malignant EGFRvIII^+^ glioma	Completed 2019	NCT01454596 (phase I/II)
HER2‐CD28 CMV‐T cells	CAR‐T Cells	IV	GBM multiforme	Completed 2018	NCT01109095 (phase I)
Autologuos Lymphoid Effector Cells	Lymphoid effector cells	NA	GBM multiforme	Completed 2013	NCT01588769 (phase I)
ALECSAT cell	NK cells, Cytotoxic T cells	IV	GBM	Completed 2013	NCT01588769 (phase I)
Allogeneic Natural Killer Cells	NK cells	Intrathecal infusion	High‐grade gliomas	Recruiting	NCT06687681 (phase II)
B7H3‐CAR‐T cells	CAR‐T Cells	Intracerebroventricular	DMG, Diffuse midline glioma, and recurrent or refractory CNS tumors	Recruiting	NCT04185038 (phase I)
CB‐NK‐TGF‐betaR2‐/NR3C1 cells	NK cells	IT	GBM	Recruiting	NCT04991870 (phase I)
Anti‐HER2 CAR macrophages	CAR macrophages	IV, IP	HER2‐overexpressing solid tumors	Active, not recruiting	NCT04660929 (phase I)
NK‐92/5.28.z + Ezabenlimab	NK cells	IC	HER2^+^ GBM	Active, not recruiting	NCT03383978 (phase I)
CART‐EGFR‐IL13Ra2	CAR‐T Cells	Intrathecal administration	EGFR‐Amplified Recurrent GBM	Active, not recruiting	NCT05168423 (phase I)
HER2‐CAR‐T cells	CAR‐T Cells	IC catheter	Pediatric CNS tumor	Active, not recruiting	NCT03500991 (phase I)
IL13Ra2‐specific CAR Tcm cells	CAR‐T Cells	IT, Intracavitary, intraventricular	Recurrent or Refractory Malignant Glioma	Active, not recruiting	NCT02208362 (phase I)
Bevacizumab + Carboplatin	Antibody	IA	Relapsed/​Refractory GBM, Anaplastic Astrocytoma	Suspended	NCT01386710 (phase I/II)
CART‐EGFRvIII cells	CAR‐T Cells	IV	First recurrence of EGFRvIII^+^ GBM	Terminated	NCT02209376 (phase I)
EGFRvIII‐CAR‐T Cells	CAR‐T Cells	CED	Recurrent GBM	Terminated	NCT03283631 (phase I)
Oncolytic adenovirus Delta‐24	Virus	IA + Mesenchymal stem cells	Recurrent high‐grade glioma	Active, not recruiting	NCT03896568 (phase I)
Oncolytic adenovirus Delta24‐RGD	Virus	CED	Recurrent GBM	NA	[236] (phase I)

Abbreviation: IT, intratumoral; IV, intravenous injection; IP, intraperitoneal injection; IA, intra‐arterial delivery; CED, convection‐enhanced delivery; CNS, central nervous system; HER2, human epidermal growth factor receptor 2; CMV, cytomegalovirus; DMG, diffuse midline glioma; GBM, glioblastoma; IL13Ra2, interleukin 13 receptor alpha 2; CAR, chimeric antigen receptor; EGFRvIII, epidermal growth factor receptor variant III; ALECSAT, autologous lymphoid effector cells specific against tumor‐cells; IDH1, isocitrate dehydrogenase type 1; TMZ, temozolomide; NA, not applicable.

CSPG4 (chondroitin sulfate proteoglycan 4), also known as NG2 (neuron‐glial antigen 2), targeted CAR‐T cells have shown promising preclinical activity across multiple solid tumors, including melanoma and glioblastoma, but remain at an early translational stage with no established clinical CAR‐T trials to date [212, 213]. CSPG4 is highly relevant in GBM due to its enriched expression in tumor cells, glioma stem‐like populations, and tumor‐associated vasculature, and is detected in 67% of GBM specimens [212]. This broad expression makes it particularly attractive for addressing both cellular heterogeneity and vascular‐supported tumor growth [213]. Such multi‐compartment expression pattern is especially significant in brain tumors, where spatial and phenotypic diversity contributes to immune evasion and therapeutic resistance [214]. Within GBM models, NG2‐directed CAR T cells effectively controlled tumor neurospheres, supported by constitutive and tumor necrosis factor‐α–inducible antigen upregulation that reduces antigen‐loss escape [212]. Collectively, these findings position CSPG4 as a promising emerging antigen for next‐generation CAR‐T engineering in glioblastoma, with broader validation across solid tumor systems reinforcing its translational potential.

Human epidermal growth factor receptor‐2 (HER2) is a well‐characterized therapeutic target, with limited expression in normal brain tissues, but overexpressed in various brain tumors, including medulloblastoma and GBM [215]. Approximately 80% of GBM cases exhibited HER2 expression, albeit with varying levels, supporting its potential for targeted therapy [215, 216, 217]. A phase I clinical trial (NCT03500991) revealed the feasibility and tolerability of HER2‐specific CAR T cells in 3 patients with anaplastic astrocytoma or ependymoma. To overcome tumor heterogeneity and antigen escape, a multiplexed administration strategy was employed to induce localized immune activation in the cerebrospinal fluid without dose‐limiting toxicity [218]. Similarly, HER2‐targeted CAR T cells showed a favorable safety profile in 17 patients with progressive GBM, with no serious adverse events (NCT01109095). In another phase I study, clinical benefits were observed in 8 patients, with a median overall survival of 11.1 months and 24.5 months from initial diagnosis. Notably, three patients remained tumor progression‐free at the last follow‐up [219].

Interleukin‐13 receptor subunit alpha‐2 (IL‐13Rα2) has also emerged as a promising target for its high prevalence in GBM [220, 221]. In a recent clinical study (NCT02208362), 65 patients with recurrent high‐grade glioma were treated with repeated IL‐13Rα2‐CAR‐T cells. This approach was demonstrated to be feasible and safe, with no dose‐limiting toxicities [222]. Importantly, combined intratumoral and intraventricular delivery elicited the strongest the strongest IFNγ‐pathway response and a significant improvement in OS (10.2 months) compared to other treatment groups [222].

Epidermal growth factor receptor variant III (EGFRvIII), a tumor‐specific antigen expressed in up to 70% of GBM cases, remains an attractive CAR T‐cell target [223]. Early clinical evaluation (NCT02209376) demonstrated the safety and feasibility of EGFRvIII‐directed CAR‐T cell manufacturing and administration [224]. Although definitive clinical benefits were not established, CART‐EGFRvIII cells successfully trafficked to brain tumors and exhibited antigen‐specific activity following intravenous infusion [224]. Besides, the immunosuppressive tumor microenvironment became more pronounced post‐treatment, highlighting a key barrier to efficacy. To overcome this limitation, combination strategies have been explored. Co‐administration of EGFRvIII‐targeted CAR T cells with the PD‐1 inhibitor pembrolizumab (NCT03726515) showed limited T‐cell expansion and persistence, with no significant clinical benefit [225]. Similarly, another trial (NCT01454596) reported transient responses, largely due to antigen escape and adaptive resistance. These findings underscore the need for improved CAR designs and combinatorial strategies to enhance therapeutic outcomes in GBM.

##### 2.4.1.1 Critical Analysis and Future Challenges

CAR T‐cell therapy has shown encouraging efficacy in brain tumors, as reflected in early‐phase clinical trial results. Nonetheless, major challenges persist in both the generation of CAR T cells and their in vivo antitumor activity, including antigen heterogeneity, complex TME, treatment‐associated neurotoxicity, and limited trafficking across the BBB. In addition, conventional ex vivo CAR T‐cell manufacturing remains a major limitation, as it is time‐consuming, costly, and impairs T‐cell function [226, 227]. The full process, which includes T‐cell isolation, genetic modification, and expansion, typically takes several weeks. This complex procedure may not suit patients with rapidly advancing cancers who require urgent treatment. On the other hand, high costs of production are incompatible with broad clinical application. To address this, mRNA‐loaded lipid nanoparticles have enabled rapid and efficient CAR expression ex vivo, offering potent antitumor activity with reduced cytotoxicity and greater flexibility than viral or electroporation‐based methods [207, 208]. Looking ahead, in vivo generation of CAR T‐cells represents an exciting future direction. Viral vectors and nanoparticles are the most widely explored platforms for delivering CAR constructs in vivo. These carriers loaded with CAR‐encoding mRNA can be prepared in advance and stored long‐term, offering an “off‐the‐shelf” product with lower costs and controllable batch‐to‐batch variability [227]. Although not yet applied in brain cancer, Hunter et al. have reported that CD8‐targeted lipid nanoparticle carrying anti‐CD19 CAR mRNA achieved tumor control in humanized mice and induced B cell depletion in cynomolgus monkeys [228]. Despite these advances, further improvements in delivery platform safety, transfection efficiency, precise T‐cells targeting, and clinical efficacy are required before widespread application in large patient populations. Collectively, these innovations may help accelerate the translation of CAR T‐cell therapy toward FDA approval for solid tumors, including glioblastoma.

#### 2.4.2 Natural Killer Cell Therapy

Considering the limitations of CAR‐T cells, there is growing interest in exploring alternative immune cell platforms, with increasing focus on NK cells. NK cells are innate cytotoxic lymphocytes with the intrinsic ability to recognize and eliminate abnormal cells [237, 238, 239]. Compared with CAR‐T cells, they offer several advantages, including a lower risk of severe toxicities, the feasibility of ‘off‐the‐shelf’ manufacturing, and natural tumor surveillance functions [237, 238].

NK cells exert cytotoxic effects through multiple pathways. They recognize tumor cells through cell surface receptors (e.g., NKG2D and CD16), natural cytotoxicity receptors (e.g., NKp44, NKp30), as well as downregulated MHC class I molecules on tumor cells, which trigger NK cell activation [240, 241, 242]. Activated NK cells then release perforin and granzymes or secrete pro‐inflammatory cytokines, including IFN‐γ and TNF‐α, leading to receptor‐mediated tumor cell apoptosis [243]. Importantly, their ability to kill tumor cells without the need for specific target antigens makes them well‐suited for highly heterogeneous and immune‐evasive tumors, such as GBM.

Unlike T cells, which are isolated from peripheral blood, NK cells can be generated from multiple sources, including embryonic stem cells, induced pluripotent stem cells, umbilical cord blood, or the commercially available NK‐92 cell line [244, 245]. This broad availability enables allogeneic ‘off‐the‐shelf’ NK cell products that could be prepared on demand, saving valuable time in patients with rapidly progressing brain cancers.

Despite appealing features, NK cell therapies face several limitations. NK cells generally exhibit limited persistence in vivo, particularly within the immunosuppressive tumor microenvironment. Their relatively poor infiltration into solid tumors further restricts long‐term therapeutic benefit [238]. To overcome these challenges, several strategies have been explored. Cytokines including interleukin‐2 and interleukin‐15 were reported to activate NK cells to prolong NK cell presence and enhance the anti‐tumor behavior [230, 231, 246]. Inspired by this, a herpes simplex virus–based oncolytic virus was engineered to express interleukin‐15/interleukin‐15 receptor alpha. This virus was demonstrated to enhance NK and CD8^+^ T cell survival and cytotoxicity in GBM models [230]. When combined with Off‐the‐shelf EGFR‐CAR NK cells, this strategy synergistically enhanced intracranial infiltration, improved CAR NK cell persistence, and significantly prolonged survival [230]. In another study, interleukin‐2 and heat shock protein 70‐treated NK cells were shown to improve NK homing and tumor growth in C6‐grafted rats [231]. Gliomas are tumors that originate from glial cells in the CNS, including the brain and spinal cord. These tumors can evade NK cell‐mediated surveillance through Gal‐1–mediated modulation of exosomal miRNAs. Moreover, Shah et al. reported that activation of TLR7 by exosomal miR‐1983 stimulated IFN‐β release and enhanced NK‐mediated cytotoxicity, suggesting a promising strategy to counteract immune evasion [247].

CAR engineering of NK cells offers additional therapeutic potential. By introducing CAR constructs, NK cells gain tumor‐associated antigen‐specific recognition that complements their innate cytotoxicity, overcomes tumor immune evasion, and amplifies their tumor‐killing capacity [248]. CAR‐NK cells targeting EGFRvIII significantly enhanced apoptosis of GBM cells in vitro [235], while GD2‐CAR NK‐92 cells demonstrated specific anti‐tumor activity against high‐GD2–expressing DMG patient‐derived cells [249]. Although NK cells alone are less effective than CAR‐T cell in fully eradicating tumors in vivo, they exhibit a substantially safer profile, with no evidence of cytokine storm or cerebral edema observed in rat models [249, 250]. The favorable safety profile of NK cells is largely attributed to their distinct cytokine secretion: activated NK cells primarily produce interferon‐gamma and granulocyte‐macrophage colony‐stimulating factor, whereas CAR T cells secrete interleukin‐1, interleukin‐6, and tumor necrosis factor‐alpha [251, 252]. Those cytokines are strongly associated with cytokine release syndrome (CRS) and severe neurotoxicity [237, 238]. Consequently, CAR‐NK therapy may offer a safer alternative for glioma treatment, particularly in paediatric DMG patients, where minimizing treatment‐related toxicity is paramount.

Encouraging pre‐clinical data of CAR‐NK cells in brain tumor have led to several ongoing clinical trials (NCT03383978, NCT04991870, NCT06687681, NCT04991870). Autologous adoptive NK and T lymphocytes were demonstrated to be safe and achieved durable responses in five patients without severe adverse effects in a phase I/IIa clinical trial (KCT0003815). A phase 1 trial (NCT01588769) used activated CD4+ T helper cells to generate autologous cytotoxic T lymphocytes and NK cells in 25 patients with recurrent GBM multiforme. Cytotoxic lymphocytes were observed homing to the tumor, with the longest recorded tumor regression lasting 27 months [253]. However, in a phase II trial (NCT02799238), the addition of Autologous Lymphoid Effector Cells Specific Against Tumor (ALECSAT) immunotherapy to radiotherapy and TMZ failed to improve PFS or OS in GBM patients [254]. Further research is required to determine whether the concurrent use of TMZ and corticosteroids adversely affects the efficacy of ALECSAT therapy.

##### 2.4.2.1 Critical Analysis and Future Challenges

Overall, NK cell‐based therapies offer a promising alternative to CAR‐T approaches in brain cancer, combining innate cytotoxicity with a favorable safety profile. CAR engineering further enhances tumor‐specific targeting and cytotoxic potency, providing a promising strategy to overcome tumor heterogeneity and immune evasion. Despite encouraging preclinical results, challenges such as limited persistence, poor tumor infiltration, and BBB hindrance restrict their clinical efficacy. Nanodelivery strategies, which have already demonstrated success in breast, colon and lung cancers, also hold promise for application in brain tumors [255, 256, 257, 258]. Emerging approaches using these platforms aim to overcome key barriers by enhancing NK cell survival, trafficking, and controlled activation within tumor sites, thereby enhancing therapeutic outcomes in glioblastoma and other aggressive brain cancers [259, 260].

#### 2.4.3 Macrophages‐Based Therapy

Despite recent advances, CAR T‐cell therapy in GBM remains limited in clinical efficacy. Clinical responses have not been durable, with tumor progression observed in two of three patients, largely due to poor T‐cell persistence [261]. The TME further constrains efficacy, with abnormal vasculature, a dense fibrotic extracellular matrix, and immunosuppressive cytokines that collectively impair CAR T‐cell function [262]. Given these challenges, attention has shifted toward alternative immune cell types such as macrophages. As abundant components of the TME, macrophages offer distinct advantages in overcoming barriers that limit CAR T‐cell therapy in GBM.

Macrophages are innate immune cells distributed throughout the body, playing vital roles in maintaining homeostasis. As powerful phagocytic cells, they clear intruding pathogens and large amounts of endogenous harmful materials to ensure normal organ function [263, 264]. Their remarkable plasticity allows them to adapt rapidly to microenvironmental signals, contributing to protective or pathogenic roles in various diseases [264, 265]. Owing to these unique features, macrophages have emerged as a promising candidate for cell therapy, particularly in tissue regeneration and cancer immunotherapy [266]. For example, human monocyte‐derived macrophages were genetically engineered to secrete a bispecific T cell engager targeting the mutated EGFRvIII, which is expressed in some subset of GBM tumors. The engineered macrophages enhanced T cell activation and effectively inhibited tumor growth [232].

CAR macrophage (CAR‐M) therapy has also been explored for treating brain cancer. Unlike CAR‐T cells, CAR‐M cells can be generated from multiple sources, including peripheral blood and induced pluripotent stem cells. They carry a lower risk of graft‐versus‐host disease and are therefore suitable for allogeneic use. Furthermore, CAR‐M cell therapy possesses unique advantages in overcoming two major obstacles in solid tumors: poor immune cell infiltration and the immunosuppressive tumor microenvironment. Macrophages, which make up to 50% of immune cells in the TME of various cancers (melanoma, renal, and colorectal cancer) [238, 267], naturally infiltrate tumors and remodel the extracellular matrix via matrix metalloproteinases [268, 269]. Given their abundance and pivotal role in the tumor microenvironment, CAR‐M therapy has been investigated in brain cancer treatment. Notably, in vivo macrophage programming offers a more streamlined and potentially cost‐effective alternative to conventional ex vivo CAR‐cell engineering. Kang et al. developed a strategy to generate CAR‐M1 macrophages in vivo using intravenously injected nanocomplexes consisting of macrophage‐targeting nanocarriers and a CAR–interferon‐γ‐encoding plasmid DNA [122]. The induced CAR‐M1 macrophages demonstrated CAR‐mediated tumor phagocytosis, immunomodulatory effects, and suppression of solid tumor growth in solid tumor‐bearing mice [122]. Enucleated mesenchymal stem cells were also applied as vehicles for targeted delivery of CAR‐encoding plasmids to glioma‐associated macrophages for in vivo CAR‐M generation. Compared with lipid nanoparticles, this platform produced sufficient numbers of CAR‐M within gliomas to achieve therapeutic efficacy against GBM [125]. To prevent tumor recurrence following surgical resection, especially from residual glioma stem cells, a cavity‐injectable hydrogel was developed [202]. This hydrogel released glioma stem cell‐targeting nanoparticles directly into the tumor cavity, enabling the in‐situ generation of CAR‐macrophages (Figure 4). These reprogrammed macrophages elicited robust antitumor immunity around the surgical site and effectively suppressed postsurgical GBM relapse in orthotopic patient‐derived mouse models [202]. Building on this approach, the same group used convection‐enhanced delivery to deliver synthetic DNA nanocarriers directly into the tumor area. This strategy reprogrammed intratumoral macrophages with tumor‐specific CARs, restoring their innate anticancer activity within the tumor microenvironment in vivo [121].

**FIGURE 4:**
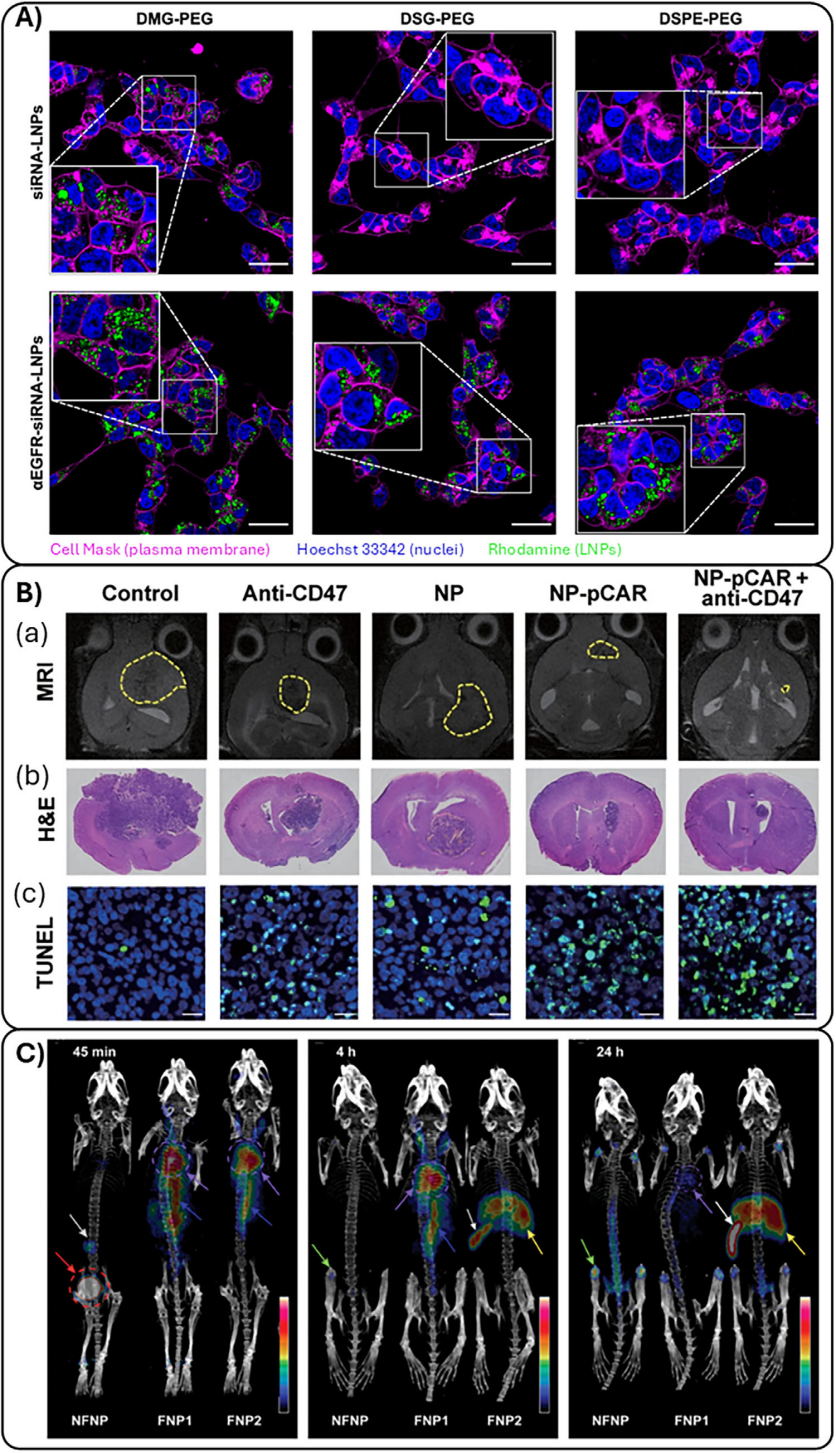
Advanced approaches to enhance the delivery, imaging, and effectiveness of biologics in brain tumor therapies. (A) Live cell confocal microscopy analysis showed the internalization of siRNA‐lipid nanoparticles in neuroblastoma SH‐SY5Y cells. Reproduced, with permission from [65]. Copyright 2024, Elsevier. DMG‐PEG siRNA‐lipid nanoparticles were more effectively taken up by SH‐SY5Y cells compared with other treatment groups. Scale bar, 30 µm. Rhodamine (green), hoechst 33342 (blue), cell mask deep red (magenta). (B) Antitumor efficacy of intratumoral Macrophage‐targeted pCAR‐laden nanoporters administration in an orthotopic intracranial GBM mouse model. Reproduced, with permission from [202]. Copyright 2022, American Association for the Advancement of Science. (B,a). In vivo T2‐weighted MRI images were obtained from glioma‐bearing mice after each treatment. Yellow dashed circles point to the tumor site. (B,b). Tumor‐bearing brain slices harvested on day 27 were subsequently imaged following hematoxylin and eosin (H&E) staining. (B,c). CAR‐macrophage–induced apoptosis was assessed in tumor‐bearing sections using terminal deoxynucleotidyl transferase–mediated dUTP nick‐end labeling (TUNEL) staining. (C) FNP1 exhibited prolonged in vivo circulation of ^89^Zr radiolabeled nanoparticles and accumulation in the brain. Reproduced, with permission from [69]. Copyright 2024, Wiley. The behaviour and pharmacokinetics of three polymer‐coated nanoparticles (FNP1, FNP2, and NFNP) were evaluated in preclinical murine models using PET/CT imaging at 45 minutes, 4 hours, and 24 hours post‐injection.

##### 2.4.3.1 Critical Analysis and Future Challenges

Macrophage‐based cellular therapies have recently entered early‐phase clinical trials for breast cancer and other solid tumors, demonstrating the capacity for tumor trafficking, modulation of the TME, and potential induction of anti‐tumor T cell immunity (e.g., NCT04660929). Although these approaches have yet to be applied in brain tumors, they hold remarkable translational potential for glioblastoma and DMG, which are constrained by dense stroma, hypoxia, and profound immunosuppression [270, 271, 272, 273]. Engineered macrophages, including CAR‐M, could be leveraged as programmable vehicles to deliver cytokines, checkpoint inhibitors, oncolytic viruses, or nucleic acids directly into the tumor, offering a strategy to reach regions in the brain that are otherwise inaccessible to conventional therapies. Beyond payload delivery, macrophages’ natural ability to cross the BBB via trans‐endothelial migration and perivascular infiltration provides a unique mechanism to bypass one of the greatest obstacles in neuro‐oncology. CAR‐M approaches, in particular, could combine tumor‐specific antigen recognition with functional reprogramming, enabling macrophages to actively engulf tumor cells, reshape the immunosuppressive microenvironment, and recruit cytotoxic T cells [274]. Integrating these strategies with existing immunotherapies or chemotherapy may overcome both physical and immunological barriers of brain tumors [275].

## 3 Current Strategies for Biologics Delivery

Efficient, rapid, and precise delivery of therapeutic agents to brain tumor sites while maintaining their therapeutic concentration remains a significant challenge in the treatment of brain cancer [277]. A range of biologic‐based therapeutic strategies, including peptide‐, protein‐, RNA‐, and drug delivery approaches, have been explored to improve targeted treatment of brain cancer (Figure 2). Drug delivery methods are broadly classified into two categories: invasive and non‐invasive administration. The choice of an appropriate drug delivery method largely depends on key physicochemical properties of the compound, such as molecular weight, solubility, stability, and lipophilicity. Additionally, the mechanism of action, the required spatial distribution, and the physiological barriers that impede delivery must be considered. These factors are critical for optimizing drug delivery strategies via different routes of administration and improving therapeutic outcomes in brain cancer treatment.

### 3.1 Invasive Delivery

Invasive delivery routes involve methods that physically penetrate the body to directly deliver therapeutic agents into the circulatory system or achieve precise, localized drug delivery, depending on the application. These approaches are particularly advantageous for bypassing physiological barriers such as the skin, intestinal epithelium, or the BBB, enabling rapid distribution of the therapeutic formulation. However, invasive routes are associated with higher risks, including potential complications such as infection or tissue damage, and generally require administration by trained medical professionals, limiting their accessibility for routine or long‐term treatment (Figure 5).

**FIGURE 5:**
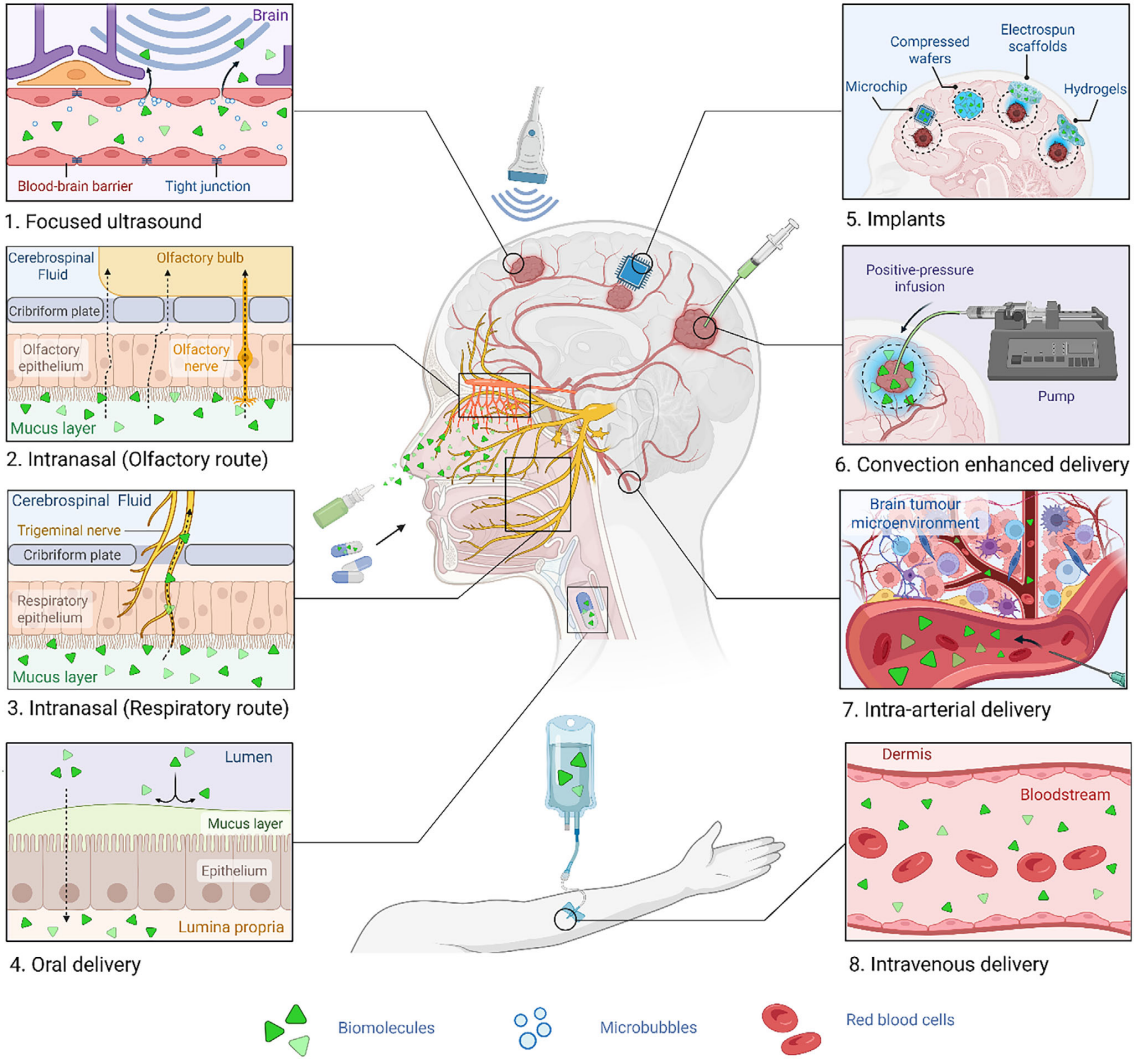
Overview of current drug administration routes and its mechanisms for the treatment of brain tumors. Panel 1: Microbubble‐mediated focused ultrasound. Microbubbles are administered intravenously and oscillate in response to focused ultrasound waves, transiently disrupting the BBB to facilitate the targeted delivery of drugs to the brain parenchyma. Panel 2: Intranasal delivery (Olfactory route). Drugs administered intranasally are absorbed through the olfactory nerve pathway located in the upper nasal cavity, bypassing the BBB and reducing systemic exposure. Panel 3: Intranasal delivery (Respiratory route). Therapeutics are absorbed through the respiratory epithelium of the nasal cavity and can reach the brain via the trigeminal nerve pathway. Panel 4: Intravenous delivery. Therapeutics are delivered directly into systemic circulation, bypassing first‐pass metabolism. Panel 5: Implants. Biologics are embedded into implantable devices, including microchips, compressed wafers, electrospun scaffolds, and hydrogels, which are surgically placed at or near the tumor site to enable localized, sustained drug release. Panel 6: Convection‐enhanced delivery. A small burr hole is created in the skull to allow surgical implantation of catheters into the brain parenchyma. Therapeutics are continuously infused under positive pressure directly into the tumor site, enabling high local drug concentration while reducing systemic exposure. Panel 7: Intra‐arterial drug delivery. A catheter is inserted into arteries supplying the brain, allowing direct infusion of therapeutics near the tumor site.

#### 3.1.1 Intra Tumor Injection

Intratumoral injection provides a promising route for localized drug delivery, particularly in treating brain tumors. By directly administering therapeutic agents to the tumor site, this method circumvents the challenges posed by the BBB and blood‐brain tumor barrier, which often impede the systemic delivery of drugs to the brain. This approach ensures a high drug concentration at the tumor site, reducing off‐target effects. Furthermore, the BBB limits the entry of therapeutic agents into systemic circulation, thereby minimizing systemic exposure and associated toxicity. Intracranial administration of bevacizumab has been shown to reduce angiogenesis‐related factors, slow tumor progression, and prolong survival compared to intravenous injection [171]. Besides, intratumoral injection offers advantages by protecting sensitive therapeutics, such as nucleic acids, from degradation in the bloodstream [36]. siRNAs targeting multiple anti‐GBM genes resulted in reduced tumor growth and burden over time following intratumoral administration [36]. This route of administration can also enhance the efficacy of cell‐based therapies. Intratumoral delivery of multifunctional mRNA‐based CAR T cells demonstrated robust anti‐glioma activity in preclinical models [47].

Despite its advantages, intratumoral injection faces limitations, including poor drug diffusion within the tumor (approximately 3–5 mm from the injection site), the need for repeated dosing to maintain therapeutic levels, and risks associated with the invasive nature of the procedure, such as bleeding and cerebral infections [17]. To address these challenges, advanced strategies like convection‐enhanced delivery, implantable drug‐impregnated polymers, and microchips have been developed [236, 278, 279, 280]. By enabling localized and precise drug delivery, intratumoral injection holds significant potential for improving treatment outcomes in brain cancer, offering a practical solution to bypass physiological barriers while minimizing systemic side effects.

#### 3.1.2 Intravenous Injection

Intravenous (IV) injection delivers therapeutic agents directly into the venous circulation, from where they are transported into the heart and subsequently distributed throughout the body via the arterial system. This method circumvents the first‐pass metabolism, ensuring rapid and accurate systemic distribution of the drug with complete bioavailability [281]. IV administration provides relatively precise control over dosage and infusion rate, making it predominantly used in the hospital setting. Intravenous drug delivery can be classified into two main approaches: bolus injection and continuous infusion [282]. IV bolus involves delivering the entire drug dose in a short period, resulting in a rapid peak plasma concentration. In contrast, IV infusion delivers the therapeutics at a constant rate over a longer duration, allowing for a gradual increase in plasma levels until a steady‐state concentration is achieved for therapeutic effect [282].

Intravenous administration remains a cornerstone for delivering therapeutic biologics in oncology, particularly monoclonal antibodies and gene therapies, as oral and alternative routes often result in reduced bioavailability and compromised stability. For instance, bevacizumab (Avastin), an anti‐angiogenesis VEGF monoclonal antibody approved for various solid tumors including recurrent GBM, is administered intravenously every two weeks to take advantage of tumor‐associated BBB disruption [283]. Similarly, immunotherapies such as nivolumab and pembrolizumab, which are checkpoint inhibitors with expanding applications in CNS malignancies, depend on IV delivery to achieve therapeutic levels in the systemic circulation, ensuring effective modulation of the immune response [284, 285].

Intravenous injection is predominantly used in hospital settings; however, the presence of the BBB significantly limits the bioavailability and concentration of therapeutic agents at the tumor site. This limitation may partially explain why IV‐administered biologics like bevacizumab (Avastin) have failed to demonstrate improvements in overall survival across several clinical scenarios. As a result, a variety of innovative drug delivery systems and alternative administration routes are being investigated to enhance the efficacy of therapeutics in brain cancer treatment. Nanoplatforms, including exosomes [123, 124], iron oxide [286], PLGA nanoparticles [64], Dendrimers [41], gold nanoparticles [194, 287], carrier proteins [288], and liposomes [289], have gained considerable attention in recent years for their ability to effectively transport therapeutic cargos across the BBB when administered intravenously.

Extracellular vesicles play an emerging role as natural drug delivery systems. Derived from cells, they are capable of transporting proteins, lipids, and nucleic acids across biological barriers, including the BBB. Their intrinsic biocompatibility, low immunogenicity, and the ability to be engineered for targeted delivery highlight their potential for brain cancer therapy [15, 290, 291]. A recent study employed exosomes derived from allogeneic bone marrow mesenchymal stem cells to deliver siRNA targeting siSTAT3 for GBM treatment [40]. Functionalized with a heme oxygenase‐1‐specific targeting peptide, the intravenous administration formulation demonstrated enhanced brain tumor accumulation. This strategy also effectively restored TMZ sensitivity, enabling a synergistic therapeutic effect in TMZ‐resistant GBM [40]. Interestingly, chemotherapy‐treated medulloblastoma cells from distinct molecular subgroups release EVs with altered miRNA profiles. These vesicles can either inhibit or promote the growth of recipient cancer cells, highlighting the complex role of EVs in modulating tumor behavior [15]. A similar BBB penetration enhancement was reported by Joshi et al., using exosomes derived from C17.2 neural stem cells that efficiently deliver protein across the in vitro BBB model by receptor‐mediated endocytosis [124].

In another study, a PAMAM dendrimer nanoparticle was decorated with the cell‐penetrating peptide tLyp‐1 and anti‐NKG2A monoclonal antibody for siRNA (siSINCT5) delivery. This targeting nanocarrier exhibited augmented BBB penetration following IV administration, resulting in an extension of median survival time and facilitated the activation of both T cells and natural killer cells within the TME in tumor‐bearing mice [41].

Gold nanoparticles play a versatile role in brain cancer therapy, serving as carriers for therapeutic agents, enabling real‐time tracking of drug delivery, and providing synergistic effects through photothermal therapy when combined with loaded drugs [292, 293]. Johnsen and colleagues investigated gold nanoparticles modified with antibodies of varying affinities for transferrin receptors to evaluate cellular uptake and BBB penetration. Interestingly, a monovalent bispecific antibody significantly improved nanoparticle uptake compared to low to intermediate levels of uptake triggered by high‐ and low‐affinity antibodies. Their results offer valuable insights into the design of nanomedicines for future brain drug delivery strategies [194].

With improved targeting capabilities, enhanced drug stability, and controlled release, these systems represent a significant advancement in addressing the challenges of conventional IV delivery. As a result, intravenous injection, combined with these innovative platforms, remains a well‐established and clinically feasible route for optimizing the pharmacokinetics and pharmacodynamics of biologics, paving the way for more effective brain cancer therapies.

#### 3.1.3 Intrathecal Delivery

Parenchymal drug delivery from the systemic circulation typically requires either transient disruption of the BBB or carrier‐mediated transcytosis. In contrast, intrathecal (IT) administration bypasses the BBB by directly delivering therapeutics into the cerebrospinal fluid, primarily via the lumbar route, providing an alternative route to access the CNS [294].

The cerebrospinal fluid (∼150 mL) circulates through the ventricular system and subarachnoid space, with a turnover of approximately 4‐5 times per day via absorption at the arachnoid villi into the venous sinuses [295]. This relatively small volume enables effective CNS exposure at substantially lower doses than required for systemic administration. Following IT injection, therapeutics distribute along the neuraxis, reaching the periventricular, cortical, and spinal cord regions [294]. Compared with systemic delivery, IT delivery exhibits distinct pharmacokinetics governed by both physicochemical properties (e.g., molecular size, charge, lipophilicity) and physiological factors such as cerebrospinal fluid flow dynamics [296]. Larger molecules, including antisense oligonucleotides, proteins, and viral vectors, primarily rely on convective cerebrospinal fluid flow due to limited diffusivity. Hydrophilic or charged molecules tend to remain confined within the cerebrospinal fluid, whereas lipophilic compounds demonstrate greater biodisponibility [297]. In addition, administration parameters, including injection volume, drug concentration, and excipients, further influence CNS distribution [298]. IT‐administered agents often exhibit prolonged half‐life in the cerebrospinal fluid compared with systemic administration, supporting sustained CNS exposure. A comprehensive understanding of the cerebrospinal fluid dynamics, interspecies differences, and regional brain distribution is critical for successful clinical translation.

IT delivery has been widely explored to deliver antibodies and mesenchymal stem cells for CNS metastases, particularly breast cancer leptomeningeal metastases [299, 300], meningeal neoplasma [301], and multiple sclerosis [302] in clinical settings. However, its application in primary brain tumors remains limited. A key constraint is the inefficient penetration of therapeutics from the cerebrospinal fluid into the brain parenchyma (typically <2 mm) [303]. As most primary brain tumors are located deep within the parenchyma, they are not effectively reached by IT‐delivered agents. Consequently, IT therapy is more effective for diseases localized within the cerebrospinal fluid space, where tumor cells are directly exposed to the drug. Furthermore, continuous CSF circulation and rapid turnover lead to drug dilution and clearance, further limiting intratumoral accumulation [294].

Despite these limitations, the unique properties of IT delivery continue to attract interest. For example, IT administration of gadobutrol has been shown to accumulate in tumor regions of a patient with an intact BBB. This method allows contrast‐enhanced MRI to guide the surgical and radiation therapy strategies [304]. In another study, IT administered red blood cell‐derived extracellular vesicles loaded with plasmid demonstrated widespread CNS distribution and efficient neuronal uptake of nucleic acids in both rodent and primate models [305]. Their results highlight the potential of a safe and effective platform for nucleic acid delivery to the CNS.

Overall, improving parenchymal penetration and controlling CSF pharmacokinetics remain key challenges for IT‐based therapies to treat brain tumors. A deeper understanding of CSF dynamics, together with advances in biologic design and delivery systems, will be essential to enhance the translational potential of IT strategies for brain cancer.

#### 3.1.4 Intra‐Arterial Delivery

Intra‐arterial (IA) drug delivery infuses the therapeutic agents directly into an artery supplying the tumor, enabling targeted regional delivery. By accessing the tumor through its feeding artery, this method achieves high local drug concentrations while minimizing systemic toxicity. IA has been widely used for brain cancer (NCT01688401, NCT02861898) and brain metastasis (NCT00362817) with chemotherapy (NCT00362817, NCT01688401), biologics (NCT01884740, NCT01811498), and stem cells (NCT03896568). Compared to IV administration, IA delivery offers several advantages, including reduced systemic drug exposure and associated adverse effects while achieving higher intratumoral drug concentrations. In contrast to intratumoral injection, IA administration allows access to deep‐seated tumors without the mechanical damage caused by needle insertion and offers potential for delivering therapeutics to metastatic disease. However, IA delivery is also accompanied by several challenges, including the obstruction of the BBB as well as reports of neurotoxicity and vascular toxicity, raising safety concerns [306]. For instance, IA infusion of TMZ caused tachycardia, hypertension, and a rash in a GBM patient. The treatment was terminated despite no detectable abnormalities on post‐infusion MRI [307]. A follow‐up study using IA TMZ infusions in healthy rats revealed cerebrovascular damage [307]. In another clinical trial investigating IA delivery of cisplatin for gliomas, severe totoxicity was observed. Among the twelve patients, nine (75%) experienced pure tone loss, with one patient becoming completely deaf, and two cases of clinically significant hearing loss [308]. These neurotoxic effects underscore the importance of carefully evaluating the safety and feasibility of therapeutic agents delivered via IA infusion, highlighting the potential advantages of biologics with a lower toxicity profile.

IA infusion also faces the challenge of BBB obstruction, and various methods have been developed to transiently permeabilize the BBB and enhance drug uptake. Pharmacological agents, such as hyperosmotic mannitol or the bradykinin analog RMP‐7, were employed to induce BBB disruption [179]. IA injection of hyperosmotic mannitol induces endothelial shrinkage and loosens tight junctions, thereby facilitating drug passage into the brain [306]. This technique has been widely used in both preclinical and clinical studies for BBB opening prior to the therapeutic agents [309, 310]. However, osmotic BBB opening followed by drug administration often results in low reproducibility and inconsistent therapeutic outcomes, influenced by factors such as mannitol concentration, injection rate, vascular anatomy and inter‐patient cerebral hemodynamics [190]. Additionally, the extensive collateralization of cerebral circulation complicates IA delivery; insufficient infusion rates may fail to achieve therapeutic effect, while excessive rates (e.g., >1 mL/ min into the internal carotid artery) can cause brain injury [190, 309]. These limitations underscore the need for more precise and controllable BBB modulation techniques to improve the safety and efficacy of IA drug delivery.

To address these challenges, dynamic susceptibility contrast MRI has been utilized to enhance reproducibility and therapeutic outcomes. Dynamic susceptibility contrast MRI facilitates precise adjustment of infusion rates to determine the perfusion territory in the brain. It also allows estimation of the blood‐brain barrier opening (BBBO) area through contrast agent perfusion mapping before mannitol injection. This ensures precise and predictable BBBO while minimizing risks. Chu and colleagues demonstrated that real‐time MRI‐guided BBBO significantly improved brain delivery of therapeutics in mice. Monoclonal antibodies, nanobodies, and polyamidoamine dendrimers showed faster penetration and greater parenchymal uptake following IA administration compared with IV delivery [190]. In a clinical setting, the same research group demonstrated successful MRI‐guided IA infusion of bevacizumab in a malignant brain tumor patient. This treatment achieved high drug accumulation, rapid neurological improvement, and tumor volume reduction without neurological deterioration [192]. The integration of preoperative MR images with real‐time angiographic perfusion images has also been investigated to enable super‐selective intra‐arterial infusion for GBM treatment. Under this perfusion‐guided approach, mesenchymal stem cells loaded with an oncolytic adenovirus Delta‐24 were targeted for delivery to the tumor area, effectively limiting non‐target infusion into potentially hazardous regions [311].

IA delivery of chemotherapies has been associated with several adverse effects, underscoring the importance of evaluating the safety profile of IA‐administered biologics to support their clinical translation. A phase I clinical trial investigated the cytotoxicity of IA cerebral infusion of cetuximab following osmotic BBB disruption with mannitol [195]. The treatment was well tolerated, with a toxicity profile comparable to IV administration and no endovascular complications. Based on these findings, a larger phase II trial evaluating efficacy and overall survival is on the way.

Overall, the integration of advanced imaging techniques with IA drug delivery facilitates precise localization of therapeutics to improve efficacy while reducing complications, positioning IA administration as a promising strategy for clinical applications in brain cancer treatment.

#### 3.1.5 Convection‐Enhanced Delivery

Convection‐enhanced delivery (CED) is a specialized technique that uses continuous, positive‐pressure infusion through stereotactically inserted catheters to distribute therapeutic agents into the CNS, circumventing the limitations of the BBB [312, 313]. Unlike diffusion‐driven methods, which rely on passive molecular movement and may lead to steep concentration gradients, CED leverages bulk flow to disperse therapeutic agents. CED maintains a positive pressure gradient for hours to days, thereby achieving more uniform and drug distribution within the targeted region of the brain parenchyma [313]. This approach has expanded the potential range of therapies for intracranial malignancies, including large macromolecules and agents characterized by poor diffusion properties or suboptimal lipophilicity. In addition, the pressure gradient established by CED can deliver drugs a few centimetres (2–3 cm) from the catheter tip [17]. This feature is critical for gliomas with a low vascular density and for preventing GBM recurrence, which typically arises within the original tumor margin.

While CED offers the advantage of achieving localized, high‐dose delivery of therapeutics with limited systemic exposure, several challenges hinder the broader clinical adoption of CED. For instance, improper catheter placement may lead to reduced treatment effectiveness and increased leakage of drugs into unintended CNS regions [312, 313]. The incidence of reflux of the drug back into the catheter is another limitation. Reflux can cause leakage and influence the drug concentration to achieve the therapeutic effect [313]. Reflux is commonly caused by air bubbles, pressure spikes during infusion, catheter insertion techniques, and the catheter design [312]. Soft and thin catheters have been shown to reduce mechanical disruption and help mitigate backflow [314]. To further address this challenge, innovations such as balloon‐tipped catheters [315], multiple‐hole catheters [316], and ultrafine catheters have been investigated with promising outcomes [317]. Despite these advancements, unpredictable complications such as infection and impairment of brain function remain concerns. Continued refinement of the catheter and the implant technologies is essential to improving the safety and efficacy of local drug delivery to the brain.

Numerous clinical trials have explored CED for delivering diverse therapeutics, including monoclonal antibodies [318], proteins (NCT02869243), chemotherapeutic drugs (NCT03154996), genes [278], viruses [236], and toxins (NCT02303678) for brain tumors, particularly GBM and DMG. A phase I trial (NCT01502917) demonstrated the safety of radiolabelled antibody [^124^I]‐8H9 delivered via CED for DMG, achieving good tumor coverage with negligible systemic exposure [319]. Another phase I clinical trial utilized CED to deliver an oncolytic adenovirus, Delta24‐RGD, for patients with recurrent GBM. Delta24‐ RGD was found to be safe and feasible in 19 patients receiving treatment, with an increased number of macrophages, CD4^+^ and CD8^+^ T cells observed post‐treatment [236]. Notably, four patients exhibited tumor response on MRI, one patient achieved complete regression and remains alive after 8 years [236]. Innovative strategies have also been investigated to maximize the potential of CED. Real‐time MRI visualization during CED administration of a monoclonal antibody with Gadolinium contrast facilitated precise tumor resection, enhancing both treatment accuracy and effectiveness [318]. However, CED has failed to demonstrate improved overall survival (OS) in a Phase III randomized trial. No survival difference was observed between CED delivered cintredekin besudotox (interleukin13‐PE38QQR) and gliadel wafers in 276 patients with recurrent GBM [320].

These findings underscore the need for further optimization of CED protocols, including improvements in catheter design, real‐time imaging guidance, and therapeutic agent optimization. Continued innovation in these areas may enhance the clinical efficacy of CED and expand its role in the treatment of brain tumors.

#### 3.1.6 Implanted Microdevices

Intratumoral injection and CED delivery represent efficient methods to bypass the BBB and deliver therapeutics directly into the brain parenchyma. However, repeated administration via invasive surgical procedures increases the risk of damage to healthy brain tissue and reduces patient quality of life [321, 322]. To address this, implantable devices have been developed, offering sustained, localized drug delivery while limiting systemic toxicity and surgical frequency. Implantable drug‐delivery systems typically feature internal reservoirs designed to store large therapeutic doses, enabling simultaneous or sequential release directly into tumor tissue. For instance, polymeric microcapsules capable of delivering up to 16 therapeutic agents and polymer microchips containing up to 36 separate reservoirs have been designed [323, 324]. After surgical implantation, drug release from these devices generally occurs through diffusion‐based mechanisms. Operators can predetermine the type and concentration of encapsulated drugs and modulate release kinetics through reservoir membrane degradation rates, pore sizes, or integration within hydrogels [323]. However, such passive devices lack active post‐implantation control over drug elution rate, dosage adjustments, or treatment duration [321, 324, 325].

To achieve more precise control over drug release, actively controlled devices have been explored, including micro‐electromechanical systems, silk microneedle patches, and bioresorbable electronic patches [326]. micro‐electromechanical systems‐based silicon microchips employ electric pulses to selectively open three drug reservoirs, providing precise control over drug volume and timing of release. When all three reservoirs were employed, up to 90% of the loaded drug could be released over 30 h [326]. However, once opened, these reservoirs cannot be resealed.

Silk microneedle patches utilize drug‐loaded, biodegradable silk fibroin to achieve independent delivery of multiple compounds [280]. By embedding plasmonic gold nanoshells at the needle tips, drug release can be actively triggered by near‐infrared irradiation, which degrades silk fibroin at a controlled rate [280]. In a recent study, a silk patch loaded with TMZ, bevacizumab, and thrombin was implanted into the brain tumor cavity of U87 glioma orthotopic models, enabling controlled release of multiple drugs at different doses [280]. Bevacizumab release was triggered by near‐infrared light two weeks post‐implantation, which inhibited neovascularization, reduced microvessel density, and improved mice survival compared to controls [280].

Bioresorbable electronic patches similarly enable actively controlled drug delivery through wireless activation using an alternating radio frequency magnetic field [279]. The incorporated magnesium‐based heating element generates mild hyperthermia, enhancing drug diffusion from oxidized starch reservoirs. The release of encapsulated therapeutics was increased by the thermal actuation regulated by the device. Raising local temperature by 5°C substantially increases drug release efficiency from 25% to 50% in the first 24 h, demonstrating adjustable and controlled therapeutic release profiles [279].

Biodiffusion chambers have been utilized in combination with cell‐based therapies, such as the Goldspire platform. These chambers possess subcellular‐sized pores size that permit the diffusion of tumor antigens and immunostimulatory molecules while preventing the escape of intact tumor cells [327, 328]. The first product of the Goldspire platform, IGV‐001, was evaluated in a phase Ib clinical trial (NCT02507583). IGV‐001 comprised ex vivo‐treated autologous GBM cells with antisense oligonucleotide (IMV‐001) targeting insulin‐like growth factor type 1 receptor and implanted abdominally. This strategy aimed to induce a tumor‐specific immune response in patients with newly diagnosed GBM patients, leading to remarkable improvement in median progression‐free survival and overall survival [327]. Encouraged by these favorable outcomes, a phase 2 clinical trial (NCT04485949) for patients with grade IV glioma is currently underway.

Another well‐known biodegradable wafer is Gliadel (carmustine), approved by the FDA in 1996 for the treatment of brain tumors, particularly GBM. Each Gliadel wafer is impregnated with the alkylating agent carmustine, and up to 8 wafers are typically implanted directly into the resection cavity following tumor removal [320]. This delivery system allows the sustained release of carmustine over approximately 20 days. Clinical trials have demonstrated improved overall survival in patients treated with Gliadel compared with placebo wafers (13.9 vs. 11.6 months) [329, 330]. However, several limitations have restricted broader clinical adoption in GBM therapy. Placement along the tumor margin limits drug penetration into deep‐seated tumor regions. The rigid polymer matrix can interfere with imaging (MRI) and conforms poorly to irregular postoperative cavities, increasing the risk of complications including cerebral edema, meningitis, and delayed wound healing [17, 331]. Wafer displacement may induce brain scarring, and ventricular obstruction can lead to obstructive hydrocephalus. In addition, small resection cavities restrict implant number and the achievable drug exposure. Despite surgical strategies enabling implantation of up to 13 wafers within the surgical bed, the clinical use of Gliadel has progressively declined [332].

Despite these technological advancements, the complex tumor microenvironment remains a significant barrier to effective drug infiltration. To improve the depth and uniformity of drug delivery, implantable pumps, including osmotic and peristaltic pumps, have been developed. Osmotic pumps utilize osmotic pressure gradients to deliver therapeutics through semipermeable membranes, while peristaltic pumps deliver drugs via peristaltic action [333, 334]. Both types feature catheters connected directly to the tumor site for precise delivery. Alzet osmotic pump is the most frequently used pump in preclinical studies. This device offers reservoir capacities ranging from 100 µl to 2 mL, with pre‐set flow rates adjustable between 0.11 and 10 µL/h [321]. Inspired by these advantages, the Alzet osmotic pump has been used for bevacizumab delivery in preclinical models [335]. Wang and colleagues used Alzet micro‐osmotic pump to deliver bevacizumab directly to the tumor site in an orthotopic xenograft mouse model [336]. Compared to intravenous delivery, CED significantly reduced microvessel density in tumor tissues. Furthermore, mice treated with CED of bevacizumab, either alone or combined with irinotecan, exhibited a 30% increase in survival than those receiving systemic administration. Despite their advantages, osmotic pumps do not allow active adjustment of infusion rates or volumes and cannot be refilled following implantation.

Peristaltic pumps, while also requiring pre‐implantation programming, offer a higher degree of control over drug release and percutaneous reservoir refilling capabilities [337]. This enhanced flexibility has facilitated their clinical translation and evaluation in human studies with robust pharmacokinetic data. SynchroMed II is an FDA‐approved, programmable implantable peristaltic pump used to treat chronic pain and spasticity. In a phase 1b clinical trial (NCT03154996), 5 patients with recurrent GBM received 48‐h intratumoral infusions of topotecan using this pump. The treatment demonstrated favorable tolerability and safety, with no grade 4 adverse events reported. The median overall survival was 12 months, and post‐treatment tissue analyses revealed significant reductions in proliferating tumor cells. These findings highlight the potential of SynchroMed II for delivering other anti‐glioma therapies.

### 3.2 Noninvasive Delivery

Noninvasive delivery routes, such as oral and intranasal administration, offer a more comfortable administration manner than invasive delivery. These strategies have gained considerable attention due to their patient‐friendly nature and suitability for repeated administration. Compared to invasive injections, non‐invasive methods reduce psychological stress and improve patient compliance, particularly during long‐term treatment. Additionally, these methods enable patients to self‐administer medications without requiring assistance from trained medical personnel, while also reducing the risk of infection, further enhancing their appeal for chronic therapeutic regimens (Figure 5).

#### 3.2.1 Intranasal Delivery

Intranasal delivery presents a promising alternative route to enhance the brain bioavailability of biologics via bypassing the BBB and directly reaching the brain parenchyma. Drugs are administered via the olfactory and trigeminal nerve pathways, where they are transported through transcellular, paracellular, and neuronal transport from the neuroepithelium of the nasal cavity to the CNS [338, 339]. This route provides a direct shortcut to the brain area, enabling fast and high brain availability of the drugs while minimizing the first‐pass metabolism and enzymatic degradation [339]. As a result, intranasal administration can reduce the systemic dose required and lower systemic toxicity. However, not all drugs are suitable for intranasal delivery, as this route requires specific physicochemical properties. Lipophilicity, molecular weight, and pKa are key factors that influence nasal absorption [340]. Lipophilic small molecules (lower than 1 kDa) like Benzodiazepines (diazepam, Valium, midazolam, etc) and certain peptides have demonstrated effective CNS delivery via the nasal route in the clinical setting [340, 341]. Conversely, hydrophilic or large biologics, such as monoclonal antibodies, may require specialized formulations to achieve adequate bioavailability. To evaluate the pharmacokinetics and biodistribution of biomolecules delivered via the intranasal route, and to assess their effectiveness in bypassing the BBB, a variety of imaging agents (^131^I, ^89^Zr, IR800, Cy5, etc.) have been tested [342]. When combined with novel formulations to enhance delivery, these agents enable effective visualization across multiple imaging modalities, including MRI, computed tomography, positron emission tomography, and focused ultrasound. However, challenges such as formulation stability, solubility, nasal clearance, and the limited absorption area of the nasal mucosa remain and require further investigation [338, 340].

To overcome these challenges, innovative drug delivery platforms such as lipid‐based nanoparticles [72], nanomicelle [62, 87, 97], PLGA nanoparticles [127], exosomes [343], and hydrogels were developed. These techniques not only enhance drug stability and permeability but also improve targeted delivery to the tumor site. For example, bevacizumab‐loaded PLGA nanoparticles were designed to improve GBM treatment. Intranasal administration to CD‐1 mice, the formulation improved brain penetration, prolonged drug residence, and increased brain bioactivity compared to free bevacizumab. Subsequently, a stronger anti‐angiogenic effect and greater tumor suppression were observed [127]. MicroRNA‐21 (miR‐21) and the receptor for advanced glycation end‐products (RAGE) contribute to GBM progression by suppressing pro‐apoptotic genes and promoting angiogenesis. MiRNA‐based therapies, however, are limited by poor stability and restricted BBB penetration. To address these limitations, self‐assembled nanoparticles combining antagomir‐21, an antisense RNA against miR‐21, with a RAGE‐antagonist peptide were developed. RAGE‐antagonist peptide served both as a carrier and as a therapeutic agent by inhibiting VEGF‐mediated angiogenesis. Intranasal delivery effectively reduced angiogenesis, suppressed miR‐21 levels, and induced apoptosis in glioma cells in vivo [97]. Cholesterol‐modified T7 nanomicelles were also explored for Slit2 siRNA delivery. Upon intranasal administration, the nanomicelles reached the brain through the olfactory route, enhancing T‐cell infiltration and remodeling the TME [75].

Intranasal administration offers a promising non‐invasive approach for delivering biologics to the brain, effectively bypassing the BBB and reducing systemic exposure. Continuous research into formulation optimization, delivery mechanisms, and targeting efficiency is essential to advance the clinical translation of intranasal delivery in the treatment of brain cancers.

#### 3.2.2 Focused Ultrasound

Approved by the FDA for the treatment of several cancers, including prostate cancer, uterine leiomyoma, and bone metastases, focused ultrasound (FUS) has attracted interest as a potential approach to improve the therapeutic outcomes in patients with GBM [344, 345, 346]. Traditional intracranial ultrasound applications have been limited due to poor penetration through the skull. Variations in skull thickness and density can result in significant attenuation, reflection, and distortion of ultrasound waves, hindering effective imaging and treatment [347]. Advances in ultrasound technology and computational methods have recently made transcranial FUS feasible for GBM patients.

FUS delivers precisely focused ultrasound beams to specific brain regions, allowing selective targeting of tumor cells while sparing adjacent healthy tissue. Two primary modalities of FUS include high‐intensity focused ultrasound and low‐intensity focused ultrasound [345, 348, 349]. High‐intensity focused ultrasound produces therapeutic effects via thermal ablation; ultrasound‐induced molecular vibrations heat the targeted tissues, causing protein denaturation, DNA fragmentation, and subsequent tumor coagulation necrosis [347]. In contrast, low‐intensity focused ultrasound primarily acts through nonthermal mechanisms, including mechanical perturbation and acoustic cavitation. Cavitation refers to the oscillation and collapse of gas bubbles induced by ultrasound compression and rarefaction cycles, with its extent influenced by ultrasound pressure, frequency, and the presence of gas bubbles [350]. Low‐intensity focused ultrasound is typically applied alongside intravenously administered microbubbles, which concentrate at the targeted site [350]. The oscillation of these microbubbles exerts stress on BBB, inducing increased cytoplasmic channels and vesicles, while reducing tight and gap junction proteins (e.g., claudins, occludins), resulting in transient BBB disruption [345, 351]. Moreover, microbubbles can locally concentrate acoustic energy to significantly reduce the ultrasound power required by up to 100‐fold, limiting off‐target effects on healthy brain tissue while enabling efficient drug delivery [352].

The precision of FUS for intracranial treatment of GBM can be significantly enhanced through integration with MRI. MRI‐guided focused ultrasound allows precise targeting of tumor tissue while sparing surrounding healthy brain regions [187, 353]. It also enables real‐time visualization of ultrasound‐induced biological effects, complemented by acoustic monitoring of microbubble emissions. Owing to its safety and reversible BBB disruption, this technique has attracted considerable interest for enhancing drug delivery in GBM therapy. Preclinical studies have successfully employed this approach to deliver free chemotherapy agents [354, 355, 356, 357], nanomedicines [358], nucleic acids [76, 98], immunotherapeutic agents such as CAR‐T cells [189], as well as novel applications including sono‐liquid biopsy [359], sonodynamic therapy [360, 361], and radiosensitization [362, 363]. For instance, 50 nm cationic lipid‐polymer hybrid nanoparticles were combined with microbubble‐enhanced focused ultrasound, achieving over a 10‐fold increase in siRNA delivery to the brain tumor microenvironment in both glioma and medulloblastoma models. Using this strategy, microbubble‐enhanced FUS delivery of Smoothened‐targeting siRNAs reduces Smoothened protein levels, resulting in significantly increased tumor cell apoptosis in vivo in an Smoothened‐activated medulloblastoma model [76].

FUS has also improved the delivery of monoclonal antibodies across the BBB. In a porcine model, microbubble‐enhanced FUS significantly increased anti‐PD‐L1 antibody delivery, resulting in a 4.8‐fold increase in MRI contrast‐enhancement volume and a 2.1‐fold increase in antibody accumulation without notable acute tissue damage [187]. Similar results were also reported by Curley et al. FUS combined with microbubbles enhanced the intratumoral dispersion of systemically administered Cy5‐labeled plasmid DNA by over 100% in both glioma and brain metastasis models [98]. Another study using FUS for delivering monoclonal antibodies targeting the CD47–SIRPα axis effectively controlled GL261 tumor growth and prolonged survival in mice, with approximately 20‐fold lower antibody dosage compared to previous administration methods [188]. In addition to transiently disrupting the BBB, FUS‐mediated delivery was also reported to enhance the penetration depth and facilitate the intranasal transport of antibodies into the brain parenchyma [364].

Moreover, this strategy can also enhance the transport and accumulation of therapeutic cells. Sabbagh et al. reported that low‐intensity pulsed ultrasound markedly improved CAR‐T cell delivery into EGFRvIII‐positive U87 glioma tumors in NSG mice, extending median survival by over 129% compared to controls without ultrasound treatment [189]. Similarly, in a GL261 glioma model, intravenous administration of CXCL10‐secreting antigen‐presenting cells (APCs) combined with low‐Intensity pulsed ultrasound significantly prolonged survival compared to all control groups. Importantly, intravenous APCs alone provided no survival benefit over PBS controls [189].

Encouraged by these promising preclinical outcomes, ongoing efforts are focused on clinical translation and further refinement of MRI‐guided FUS‐based strategies. Current clinical studies have demonstrated the feasibility of FUS‐enhanced delivery for chemotherapy drugs, including temozolomide [356], paclitaxel [354], carboplatin [355], doxorubicin [357]. However, clinical translation of FUS‐enhanced biologics for GBM treatment remains slow, and cases are limited. In a single‐arm open‐labelled phase I study (NCT03714243), MR‐guided focused ultrasound was used to deliver monoclonal antibody trastuzumab in four patients with Her2‐positive brain metastasis. MR‐guided FUS remarkably augmented drug uptake, with increased accumulation observed in 87% ± 17% of sonicated voxels, and up to a 450% increase in voxel‐wise enhancement. Following treatment, unidimensional tumor measurements decreased by 19% ± 12% after treatment [180]. These results further underscore the potential clinical benefit and translational feasibility of FUS for GBM treatment.

#### 3.2.3 Oral Delivery

Oral administration is the most popular route of small‐molecule drug delivery due to its convenience, patient compliance and non‐invasive nature. Temozolomide remains the cornerstone of oral chemotherapy for high‐grade gliomas and is widely regarded as the standard‐of‐care alkylating agent in neuro‐oncology, particularly for glioblastoma and anaplastic astrocytoma. Temozolomide's favorable oral bioavailability, ability to cross the BBB, and demonstrated survival benefit in combination with radiotherapy (the Stupp protocol) have established it as the clinical benchmark for malignant glioma treatment [365]. However, its efficacy across brain tumor types remains limited, and outcomes are strongly influenced by MGMT promoter methylation status, with resistance representing a major barrier in high‐grade gliomas. These limitations highlight a critical translational gap: while temozolomide demonstrates that oral administration can achieve clinically meaningful brain tumor exposure, current therapeutic efficacy remains constrained by drug resistance and insufficient tumor specificity. This has driven the development of next‐generation oral delivery systems aimed at improving stability, absorption, and tumor‐targeted transport across both the gastrointestinal, and the BBB.

While oral administration remains the dominant approach to small‐molecule drug delivery, applying this route to biologics poses significant challenges. Some biologics, particularly macromolecules, experience poor transport across the intestinal membrane, limiting absorption into systemic circulation. In addition, biologics are susceptible to enzymatic degradation in the gastrointestinal tract and the systemic circulation, leading to low bioavailability. To address the challenges associated with oral biologics delivery, various strategies have been developed, including the use of permeation enhancers, nanoparticles, peptide modifications, intestinal patches. and mucoadhesive modifications [366, 367]. Recent FDA approvals of oral peptide drugs including semaglutide (2019 **Rybelsus**), semaglutide (2026 **Wegovy**), octreotide (2020 **Mycapssa**), and icotrokinra (2026 **Icotyde^TM^**) highlight the growing role of permeation enhancers and advanced formulations in enabling oral delivery of peptide therapeutics. Oral delivery of biologics such as peptides (NCT03051009), vaccines (NCT03220737), and proteins (NCT01287195) is thus being clinically investigated for various diseases, including diabetes (NCT02827708), tuberculosis (NCT00396370), typhoid (NCT02391909), and acromegaly (NCT02685709). Despite advancements in overcoming intestinal barriers and enhancing stability, crossing the BBB to effectively target brain tumors without compromising therapeutic efficacy remains a critical obstacle, resulting in limited success. A recent study developed an antisense oligonucleotide‐encapsulated lipid polymer micelle system, modified with Angiopep‐2 and TAT for glioma targeting. Their micelles showed enhanced uptake by LN229 glioma cells in vitro, inducing apoptosis and inhibiting cell proliferation. Oral administration in an orthotopic glioma xenograft mouse model activated pro‐apoptotic proteins p53 and Caspase‐3, resulting in apoptosis, thereby suppressing tumor progression and extending nude mice survival [96].

### 3.3 Comparison Between Noninvasive and Invasive Delivery

In conclusion, selecting an appropriate delivery strategy is critical for achieving therapeutic efficacy in brain cancer. Key factors include the physicochemical properties of the biologic, its mechanism of action, the desired spatial distribution, and the need for repeated dosing. Biologics are inherently unstable and prone to degradation, which often necessitates invasive administration to ensure sufficient stability and efficacy.

Invasive strategies, such as CED and IA infusion, bypass biological barriers and metabolic clearance to achieve high local drug concentrations. CED maintains a positive pressure gradient within the brain parenchyma, enabling uniform distribution of macromolecules with poor diffusivity or suboptimal lipophilicity [312, 313]. Similarly, IA administration facilitates access to deep‐seated tumors and metastatic lesions while reducing mechanical injury by needle insertion. Implantable microdevices further expand the potential of invasive approaches by housing internal reservoirs that allow sustained, localized release while minimizing systemic toxicity and surgical frequency [321, 323]. Despite these advantages, invasive delivery still carries notable risks, including infection, tissue damage, and the need for highly trained medical personnel. In addition, repeated dosing can impair patient quality of life, limiting their feasibility for long‐term therapy.

Non‐invasive strategies, by contrast, offer patient‐friendly alternatives that support repeated administration and self‐management, thereby improving compliance and reducing infection risk. Oral delivery remains the most common route but faces multiple challenges, including first‐pass metabolism, limited stability in systemic circulation, poor penetration across BBB, and inefficient tumor internalization. Although penetration enhancers and formulation strategies can improve absorption, preserving structural integrity and bioactivity throughout this complex journey remains difficult. Intranasal delivery provides a more direct route to the CNS, bypassing the gastrointestinal tract and BBB while reducing systemic toxicity [338, 339]. This method enables rapid brain uptake of lipophilic small molecules and certain peptides, although large molecules and hydrophilic compounds, such as monoclonal antibodies, generally require specialized formulations or carriers to achieve therapeutic levels [340]. Other noninvasive approaches, such as FUS‐mediated BBB opening, are emerging as promising modalities to transiently enhance brain permeability and expand the range of deliverable biologics [345].

Another important consideration is the anatomical location and biological characteristics of each tumor type, which critically determine the feasibility and efficacy of therapeutic delivery. High‐grade astrocytomas, including GBM, are typically infiltrative and located within the brain parenchyma [10, 17, 368]. For these tumors, FUS has been widely explored to transiently disrupt the BBB and enhance drug penetration. While CED or implantable microdevices enable localized administration into the tumor core and infiltrating margins, achieving sufficient intratumoral concentrations while minimizing systemic toxicity. Furthermore, GBM exhibits diverse heterogeneity and a highly immunosuppressive TME. These features make GBM suitable for monoclonal antibodies, CAR‐T cell therapies, or RNA‐based therapeutics that interact with surface receptors or modulate gene expression post‐transcriptionally [33, 191, 193, 204]. To overcome the immunosuppressive TME, macrophage‐based therapies, particularly engineered CAR‐M, have shown promise by enhancing phagocytosis, antigen presentations, and immune reprogramming within the TME [121, 122, 202]. In contrast to GBM, meningiomas, which are generally extra‐axial and surgically accessible, can be treated via direct intratumoral injection or implantation at the resection site, making invasive delivery more practical [369, 370]. For tumors located in surgically inaccessible or delicate regions, such as DMG within the brainstem, CED is better suited to enhance drug diffusion. Targeting surface antigens (e.g., HER2, B7‐H3) or blocking oncogene expression (e.g., H19, AF4/FMR2 4) has shown promising therapeutic potential in DMG [77, 319, 371]. Although intra‐arterial and intranasal approaches offer alternative routes, results remain largely preclinical.

Overall, aligning delivery strategies with tumor location and accessibility is essential to balance therapeutic efficacy, safety, and patient quality of life, highlighting the need for precision delivery approaches tailored to the spatial and pathological context of individual brain tumors.

## 4 Future Direction and Clinical Translation

Biologic materials are central to emerging strategies for brain cancer therapy due to their programmable specificity, molecular precision, and capacity for immune modulation. Monoclonal antibodies represent the most clinically mature class, supported by robust structural stability and well‐established targeting behavior. In parallel, CAR‐T cell therapies have demonstrated powerful anti‐tumor activity through precise recognition of tumor‐associated antigens and potent immune activation [274, 372]. However, their clinical impact remains fundamentally constrained by one dominant barrier: ineffective delivery across the BBB, leading to poor tumor access, systemic toxicity, and limited intracranial persistence [373, 374].

Overcoming the BBB requires a shift from biologics alone to integrated material‐enabled delivery systems that govern access to the brain. Effective translation therefore depends on the rational design of materials capable of modulating permeability, transport, and retention within the central nervous system. Rather than functioning as passive carriers, emerging biomaterials are increasingly engineered through tuneable size, surface chemistry, charge distribution, deformability, and ligand‐mediated transport mechanisms to actively interact with BBB physiology [375, 376]. Permeation‐enhanced strategies, including receptor‐mediated transcytosis, adsorption‐mediated transport, and transient BBB modulation, have become central approaches for improving intracranial accumulation of biologics while minimizing systemic toxicity [51, 196, 229, 377]. Stimuli‐responsive and externally actuated materials, particularly ultrasound‐responsive nanoparticles and microbubble‐assisted systems, enable localized and reversible BBB opening to enhance delivery precision and therapeutic penetration into tumor tissue [98, 378, 379, 380]. Engineered exosomes, multifunctional nanoparticles now define a new class of delivery materials designed specifically to navigate biological barriers and localize therapeutics within brain tumors. Physical delivery platforms—including ultrasound‐mediated BBB disruption [381, 382, 383], magnetic targeting systems [384, 385], and implantable microdevices [386]—provide precise spatial and temporal control over intracranial delivery. Together, these systems transform delivery from a passive pharmacokinetic constraint into an actively engineered function of materials design. Importantly, they extend beyond conventional biologics, enabling controlled delivery of next‐generation modalities such as mRNA therapeutics, CRISPR‐based gene editors, and engineered cytokines, thereby expanding the therapeutic design space for brain cancer.

Despite these advances, over 90% of oncology therapeutics that succeed in animal models fail in human clinical trials due to toxicity or lack of efficacy [387]. This failure reflects not incremental limitations but structural inadequacy in conventional preclinical systems. As a result, human‐relevant validation strategies have become essential components of the translational pipeline. Human‐relevant model systems are increasingly integrated into the development of brain cancer therapies to address the limitations of conventional preclinical testing [388, 389]. Rather than functioning as independent experimental platforms, organoids, bioprinted tumor constructs, and organ‐on‐chip and artificial intelligence systems collectively serve as predictive environments for evaluating biologic materials and their delivery performance [373, 374, 390, 391, 392, 393]. This shift enables direct assessment of clinically relevant parameters, including BBB penetration, intratumoral distribution, and therapeutic response under physiologically realistic conditions [394]. Within this framework, patient‐derived tumor models provide a biologically faithful representation of tumor heterogeneity and enable evaluation of how biologic therapeutics and their associated delivery systems behave in patient‐specific disease contexts. This is particularly critical in brain cancer, where spatial and molecular heterogeneity strongly influences therapeutic response and contributes to treatment resistance [394]. Importantly, the primary value of these systems lies in their ability to connect material design to functional biological outcome. By providing a controlled and human‐relevant testing environment, they enable systematic refinement of delivery systems based on observed performance, thereby establishing a direct feedback loop between biomaterial engineering and therapeutic efficacy [394]. In this way, they function as essential validation interfaces that de‐risk translation rather than as standalone technological directions.

Collectively, these validation systems reduce reliance on poorly predictive animal models and substantially improve the reliability of clinical translation. In parallel, regulatory frameworks are shifting toward human‐relevant testing paradigms for therapeutic development [388, 389]. Taken together, the development of biologic therapies for brain cancer follows a clearly defined translational pathway: biologic design, material‐enabled delivery, human‐relevant validation, and clinical application. This pathway depends on the tight integration of materials engineering, delivery system design, and predictive validation platforms. Materials science therefore functions as the central and non‐substitutable driver of therapeutic translation in brain cancer.

## 5 Conclusion

The future of brain cancer therapy will be shaped by the integration of advanced materials with precision delivery strategies that can navigate the blood–brain and tumor microenvironments. Engineered platforms that mimic physiological barriers and tumour dynamics allow for real‐time assessment of therapeutic behavior, overcoming the limitations of conventional in vitro and animal models. By combining these predictive models with modular, tuneable materials, researchers can design systems that interact dynamically with tumors, enhancing targeting, efficacy, and safety. This convergence of materials innovation, delivery engineering, and physiologically relevant modelling represents a paradigm shift—transforming brain cancer treatment from a largely empirical endeavor into a rationally designed, highly precise, and clinically translatable strategy.

## Conflicts of Interest

The authors declare no conflicts of interest.

## Data Availability

The data that support the findings of this study are available from the corresponding author upon reasonable request.
